# DNA barcoding herbaceous and woody plant species at a subalpine forest dynamics plot in Southwest China

**DOI:** 10.1002/ece3.4254

**Published:** 2018-06-25

**Authors:** Shao‐Lin Tan, Ya‐Huang Luo, Peter M. Hollingsworth, Kevin S. Burgess, Kun Xu, De‐Zhu Li, Lian‐Ming Gao

**Affiliations:** ^1^ Key Laboratory for Plant Diversity and Biogeography of East Asia Kunming Institute of Botany Chinese Academy of Sciences Kunming, Yunnan China; ^2^ Germplasm Bank of Wild Species Kunming Institute of Botany Chinese Academy of Sciences Kunming, Yunnan China; ^3^ College of Life Sciences University of Chinese Academy of Sciences Kunming, Yunnan China; ^4^ Royal Botanic Garden Edinburgh Edinburgh UK; ^5^ Department of Biology College of Letters and Sciences Columbus State University University System of Georgia Columbus Georgia; ^6^ Lijiang Forest Ecosystem Research Station Kunming Institute of Botany Chinese Academy of Sciences Lijiang China

**Keywords:** DNA barcoding, forest dynamics plot, herbaceous species, species identification, woody species, Yulong Mountain

## Abstract

Although DNA barcoding has been widely used to identify plant species composition in temperate and tropical ecosystems, relatively few studies have used DNA barcodes to document both herbaceous and woody components of forest plot. A total of 201 species (72 woody species and 129 herbaceous species) representing 135 genera distributed across 64 families of seed plants were collected in a 25 ha CForBio subalpine forest dynamics plot. In total, 491 specimens were screened for three DNA regions of the chloroplast genome (*rbcL*,* matK*, and *trnH*‐*psbA*) as well as the internal transcribed spacers (ITS) of nuclear ribosomal DNA. We quantified species resolution for each barcode separately or in combination using a ML tree‐based method. Amplification and sequencing success were highest for *rbcL*, followed by *trnH‐psbA*, which performed better than ITS and *matK*. The *rbcL* + ITS barcode had slightly higher species resolution rates (88.60%) compared with *rbcL* + *matK* (86.60%) and *rbcL + trnH‐psbA* (86.01%). The addition of *trnH‐psbA* or ITS to the *rbcL + matK* barcode only marginally increased species resolution rates, although in combination the four barcodes had the highest discriminatory power (90.21%). The situations where DNA barcodes did not discriminate among species were typically associated with higher numbers of co‐occurring con‐generic species. In addition, herbaceous species were much better resolved than woody species. Our study represents one of the first applications of DNA barcodes in a subalpine forest dynamics plot and contributes to our understanding of patterns of genetic divergence among woody and herbaceous plant species.

## 1 INTRODUCTION

Accurate identification of plant species is a key component of biological, ecological, and evolutionary studies. Standard morphological techniques can be ineffective, especially for large and complex taxa (Liu et al., 2017; Percy et al., 2014; Yan et al., 2015), particular life stages (Gonzalez et al., 2009), or when only plant fragments are available (Jones et al., 2011; Kesanakurti et al., 2011). To support morphological identification, DNA barcoding has become widely used for the identification and confirmation of plant species (Fazekas et al., 2008; Hollingsworth et al., 2009; Kress & Erickson, 2007; Kress, Wurdack, Zimmer, Weigt & Janzen, 2005; Li et al., 2011). The plastid DNA regions *rbcL, matK, trnH‐psbA* and the internal transcribed spacers (ITS) of nuclear ribosomal DNA have previously been used in systematic studies at various taxonomic levels (Azani et al., 2017; Baldwin et al., 1995; Chase et al., 1993; Harrington, Edwards, Johnson, Chase & Gadek, 2005), as well as for the taxonomy and species identification of specific target families and genera (Liu, Moeller, Gao, Zhang & Li, 2011; Xu et al., 2015; Yan et al., 2015). Increasingly, surveys of local (Kress et al., 2009) and regional (Burgess et al., 2011; De Vere et al., 2012; Liu, Yan et al., 2015) floras are based on DNA barcoding with these regions.

One key step in the DNA barcoding process is the establishment of a reliable and comprehensive database (reference library) that includes both high‐quality DNA barcode sequences and accurately identified voucher specimens (Taberlet, Coissac, Pompanon, Brochmann & Willerslev, 2012). Once established, such databases can become a valuable toolkit for molecular ecologists to identify plant material such as pollen, seeds, seedlings, roots, and even bark fragments (Liu et al.,^2018^). DNA barcoding is now becoming essential for a broad range of ecological applications, such as biodiversity assessments (Lahaye et al., 2008; Yoccoz et al., 2012), establishing pollination networks (Bell et al., 2016; Vamosi, Gong, Adamowicz & Packer, 2017), diet analysis (Kartzinel et al., 2015; Valentini, Pompanon & Taberlet, 2009), and determining the mechanisms underlying community assembly (Kress et al., 2009, 2010; Pei et al., 2011).

During the last three decades, CTFS‐ForestGEO (http://www.forestgeo.si.edu/), a worldwide network of over 60 long‐term forest dynamics plots (FDPs), has been established for monitoring of community dynamics in forest ecosystems and investigation of community assembly mechanisms (Anderson‐Teixeira et al., 2012). To date, millions of trees representing over 10,000 species have been tagged, mapped, and identified. The application of well‐resolved phylogenies based on the DNA barcode sequences of species within these communities is substantially improving our understanding of community assembly mechanisms (Kembel & Hubbell, 2006; Kress et al., 2009; Webb, 2000; Yang et al., 2014). However, although there are over 60 plots in the CTFS‐ForestGEO network, only a few plots have genetic libraries (Kress et al., 2009; Pei et al., 2015) or community phylogenies (Erickson et al., 2014; Kress et al., 2009, 2010; Pei et al., 2011) based on DNA barcodes.

Herbaceous plants contribute substantially to the diversity and abundance of forests ecosystems, particularly in temperate regions (Gilliam, 2007, 2014; Luo et al., 2016a, 2016b; Wang, Tang & Fang, 2007). However, interactions between herbaceous and woody plant layers in community assemblages have long been neglected in forest dynamics models, even though it is widely known that herbaceous species can impact the regeneration of tree species (Murphy, Salpeter & Comita, 2016; Thrippleton, Bugmann, Kramer‐Priewasser & Snell, 2016). DNA barcoding has mainly been used for tree species in forest dynamic plots of tropical, subtropical, and temperate regions (Erickson et al., 2014; Kress et al., 2009; Pei et al., 2015). Thus in many cases, the herbaceous component of the community remains underexplored. In this study, we use DNA barcodes to identify and document the herbaceous and woody flora of a 25‐ha subalpine spruce‐fir forest dynamics plot on the Yulong Mountain, northwestern Yunnan, China. We aim to address the following three questions: (a) What barcode strategy provides the highest species resolution in a subalpine forest? (b) Do species discrimination rates vary among herbaceous and woody species in this community? (c) Does the number of species within a genus affect species resolution rates in high elevation forest dynamic plots?

## 2 MATERIALS AND METHODS

### 2.1 Study site and sampling

Yulong Mountain (26°58′–27°18′ N, 100°04′–100°16′ E) belongs to the Himalaya–Hengduan Mountains Region, which is a part of a global biodiversity hotspot in the Mountains of Southwest China; over 2,800 species of seed plants have been recorded from this mountain (Liu, Li et al., 2015). This study targets a 25‐ha (500 m × 500 m) forest dynamics plot that is located in the subalpine region of the east side of Yulong Mountain. The plot was established between 2012 and 2014 according to standard protocols established by the CTFS‐ForestGEO network (Huang et al., 2017): all trees with a diameter at breast height (DBH) ≥1.0 cm were tagged, identified, measured, and mapped according to these protocols. The altitude of the plot ranges from 3220 m to 3344 m, it is by far the highest forest dynamics plot established to date.

Between 2013 and 2015, a total of 491 specimens (201 species, 135 genera, and 64 families of seed plants) were sampled for the recovery of DNA barcodes (Supporting Information Table S1), and each species were represented by two to six individuals except for nineteen rare species with a single individual sampled. Our samples included 129 herbaceous species and 72 woody species. All DNA vouchers were deposited in the Herbarium of Kunming Institute of Botany (KUN), Chinese Academy of Sciences, Yunnan, China.

### 2.2 DNA extraction, amplification, and sequencing

Total genomic DNA was extracted from silica‐dried leaf tissue using a modified CTAB method (Doyle, 1987). The DNA was diluted with TE buffer to a final concentration of 10–50 ng/μl for PCR amplification. We amplified the *rbcL*,* trnH‐psbA*,* matK*, and ITS (ITS for Angiosperms; ITS2 for Gymnosperms) gene regions based on the primers and PCR conditions outlined in Supporting Information Table S2 following Gao et al. (2012). PCR mixtures (20 μl) contained 10–50 ng template DNA, 2.5 μM forward and reverse primer each, and 10 μl 2 × Taq PCR Master Mix (TIANGEN). PCR amplification was performed using a GeneAmp PCR System 9700 thermal cycler (PerkinElmer, Foster City, CA, USA). PCR products were purified using ExoSAP‐IT (GE Healthcare, Cleveland, OH, USA). Using the same primers as those used for PCR, bidirectional sequencing reactions were carried out on an ABI 3730 DNA Sequencer (Applied Biosystems, Foster City, USA) in the biology laboratory of the Germplasm Bank of China, Kunming Institute of Botany, Chinese Academy of Sciences. Some of the PCR products were sent to Tsingke Biological Technology Co., Ltd, Beijing for purification and sequencing. All sequences were deposited in GenBank (accession numbers are listed in Supporting Information Table S6).

### 2.3 Data analysis

Raw sequences were assembled and manually edited in Geneious (version 4.8.5). Consensus sequences were generated and aligned using ClustalW with default parameter settings and adjusted manually where necessary. All *rbcL* and *matK* sequences could be readily aligned but *trnH‐psbA* and ITS sequences had to be partitioned and aligned separately by family, order or closely related orders: in total 24 separate alignments were created and then concatenated with the *rbcL* and *matK* alignments (Kress et al., 2009).

Compared with other methods for DNA barcoding analysis, the tree‐based method has the advantage of providing a graphical representation of species divergence (Hebert, Stoeckle, Zemlak & Francis, 2004). In this study, Maximum Likelihood (ML) trees were used for testing the efficiency of DNA barcodes for species resolution. The ML trees based on the GTR + GAMMA model with 1,000 bootstrap replicates were reconstructed in RAxML via CIPRES (http://WWW.phylo.org/). A species was considered as being correctly resolved when all the individuals of the same species formed a monophyletic group with a bootstrap value over 50%. We assessed variation in percentage species resolution rates for *rbcL* and *matK* barcodes separately, and in combination (*rbcL *+ *matK* [RM], *rbcL *+ *trnH‐psbA* [RP], *rbcL *+ ITS [RI], *rbcL *+ *matK *+ *trnH‐psbA* [RMP], *rbcL *+ *matK *+ ITS [RMI], and *rbcL *+ *matK *+ *trnH‐psbA + *ITS [RMPI]. We also assessed rates of species resolution among herbaceous species and woody plant species for the same barcode combinations. Differences in species resolution rates among genera with varying numbers of species per genus (1–5) and the type of barcode combination were compared using a Poisson distribution of a generalized linear model in the R package *multcomp*. We also performed a simple linear regression to detect whether species resolution rates were significant negatively correlated with species/genus ratio using the data obtained from floristic DNA barcoding studies at plot and regional scales. All analyses were implemented in R software (R Core Team 2016).

## 3 RESULTS

For gymnosperms, we obtained high‐quality bidirectional sequences for the *rbcL*,* matK*, and *trnH‐psbA* gene regions of the chloroplast genome; we were able to obtain sequences for the ITS region from all but one sample (Supporting Information Table S3). For angiosperms (Supporting Information Table S3), PCR and sequencing success of the *rbcL* gene region were the highest (99.37% and 98.74%, respectively). PCR success of *trnH‐psbA* was also high (97.05%), but sequencing success was lower (92.02%). Substantially more effort was required to recover ITS sequences, which had relatively high PCR success (96.01%) but sequencing success (89.50%) for ITS was the lowest among the four gene regions, largely due to fungal contaminants. Three pairs of primers were required for the recovery of *matK*, but PCR and sequencing success were relatively low for this gene region (93.07% and 92.02%, respectively).

**Figure 1:**
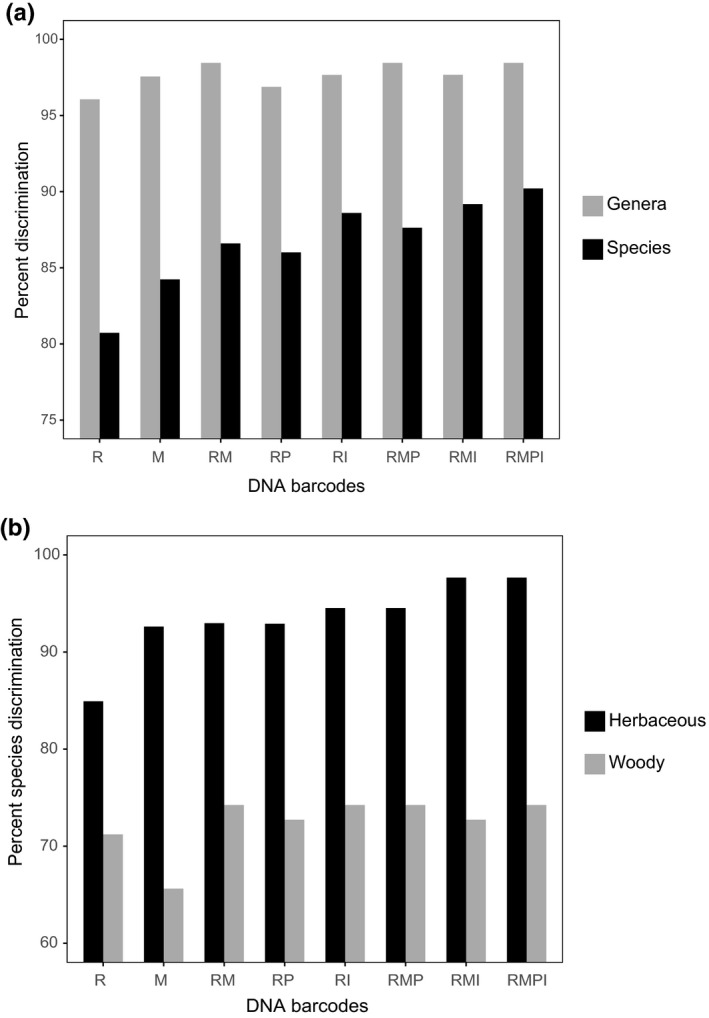
Discrimination success of DNA barcodes and their combinations for (a) Species and genera of angiosperms and (b) Herbaceous and woody species, in a high elevation subalpine plot on Yulong Mountain, Yunnan, Northwest China. We assessed variation in percentage discrimination for *rbcL* [R] and *matK* [M] barcodes separately, and in combination (*rbcL *+ *matK* [RM], *rbcL *+ *trnH‐psbA* [RP], *rbcL *+ ITS [RI], *rbcL *+ *matK *+ *trnH‐psbA* [RMP], *rbcL *+ *matK *+ ITS [RMI], *rbcL *+ *matK *+ *trnH‐psbA +* ITS [RMPI]

All gymnosperms (six species belonging to six genera) were correctly resolved using the four markers separately. For angiosperms, species resolution was higher for *matK* (84.24%) compared to *rbcL* (80.73%; Supporting Information Table S4; Figure 1a). The combination of the two chloroplast coding regions (RM) resolved 86.60% of angiosperm species, similar to RP (86.01%), and slightly lower than RI (88.60%). Adding the supplementary barcodes *trnH‐psbA* or ITS to RM only slightly increased percentage species resolution (RMP 87.63%; RMI 89.18%), although the four barcodes in combination (RMPI) achieved the highest rate of species resolution (90.21%; Supporting Information Table S4; Figure 1a).

Overall resolution rates of herbaceous species tended to be higher than that of woody species for any barcode or barcode combination (Supporting Information Table S4; Figure 1b). For the identification of herbaceous species, *matK* distinguished a higher proportion (92.62%) of species than *rbcL* (84.92%). In combination, species resolution rates for RM (92.97%), RP (92.91%), RI (94.53%), and RMP (94.53%) were higher than any single barcode. RMPI (97.66%) and RMI (97.66%) had the highest values (Supporting Information Table S4; Figure 1b). Species resolution rates of woody species were lower for all barcodes, which ranged from 65.63% (*matK*) to 74.24% (RM, RI, RMP, and RMPI), compared to herbaceous plants, which ranged from 84.92% (*rbcL*) to 97.66% (RMI and RMPI). Species resolution rates between herbaceous species and woody species for R, RM, RP, RI, and RMP, were lower for woody species by 13.71%, 18.73%, 20.18%, 20.29%, and 20.29%, respectively. Woody plants had much lower values for other combinations of gene regions, differences between these two groups of taxa were greatest for RMPI (23.42%) and RMI (24.93%). The single greatest difference in species resolution rate between herbaceous species and woody species was for *matK* (26.99% lower for woody species). Although adding *trnH‐psbA* or ITS to the core barcode RM did not improve resolution among woody species it did improve the resolution of herbaceous species. The improvement of species discrimination was greater by adding ITS (RMI, 97.66%) than by adding *trnH‐psbA* (RMP, 94.53%) for herbaceous plants (Supporting Information Table S4; Figure 1b).

**Figure 2:**
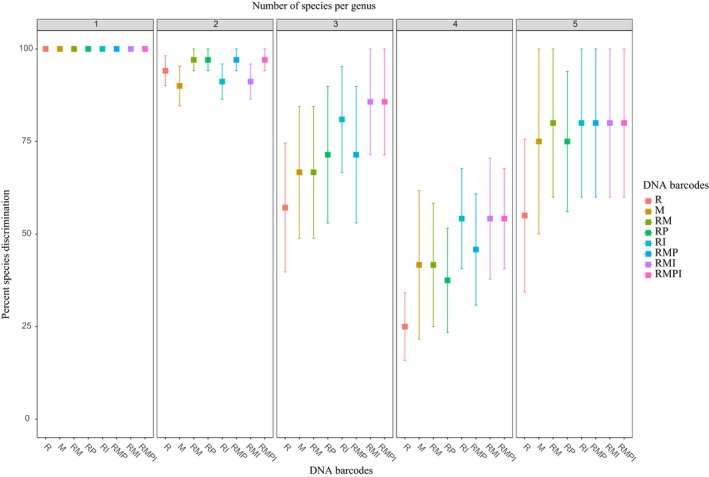
Percentage species discrimination among genera with different numbers of species in a high elevation subalpine plot on Yulong Mountain, Yunnan, Northwest China. We assessed variation in percentage species discrimination for *rbcL* [R] and *matK* [M] barcodes separately, and in combination (*rbcL *+ *matK* [RM], *rbcL *+ *trnH‐psbA* [RP], *rbcL *+ ITS [RI], *rbcL *+ *matK *+ *trnH‐psbA* [RMP], *rbcL *+ *matK *+ ITS [RMI], *rbcL *+ *matK *+ *trnH‐psbA +* ITS [RMPI]. Numbers along top of figure represent genera that vary in the number of species per genus (1–5)

Percent species resolution varied among genera and ranged from 0–100% (Supporting Information Figure S1). Single barcodes *rbcL* and *matK*, as well as any barcode combination were able to distinguish all species in genera represented by single species (Table 1; Figure 2). Genera with two species had the highest percent species resolution rates among polytypic genera, ranging from 90% (*matK*) to 97.06% (RM, RP, RMP, and RMPI). The resolution rate of any barcode or barcode combination was lower when the number of species per genus increased to three species per genus, which ranged from 57.14% (*rbcL*) to 85.71% (RMI and RMPI). Genera with four species showed the lowest level of species discrimination, which ranged from 25% (*rbcL*) to 54.17% (RI, RMI, and RMPI). Genera with five species per genus were better resolved than that of genera with four species, but were less resolved than genera with one and two species. In genera with five species, *rbcL* alone resolved 55% of the species, *matK* and RP resolved 75%, and other barcode combinations resolved 80% of the species (Table 1; Figure 2).

**Table 1:** Percentage species discrimination using DNA barcodes for genera that vary in the number of species per genus (S/G) in the Yulong Mountain Forest Dynamics Plot (YFDP), Northwest Yunnan, China

Barcode	S/G = 1 (%)	S/G = 2 (%)	S/G = 3 (%)	S/G = 4 (%)	S/G = 5 (%)
R	100	94.12	57.14	25.00	55.00
M	100	90.00	61.11	41.67	75.00
RM	100	97.06	66.67	41.67	80.00
RP	100	97.06	71.43	37.50	75.00
RI	100	91.18	80.95	54.17	80.00
RMP	100	97.06	71.43	45.83	80.00
RMI	100	91.18	85.71	54.17	80.00
RMPI	100	97.06	85.71	54.17	80.00

The generalized linear model analysis indicated that DNA barcode regions (*df* = 7, χ^2^ = 15.19; *p* = 0.0336), as well as the number of species per genus (*df* = 4, χ^2^ = 2,254.29; *p* < 0.001), have a significant effect on the species resolution rate. The interaction between DNA barcodes and the number of species per genus was also significant (*df* = 28, χ^2^ = 201.97; *p* < 0.001). Significant differences in percentage species resolution among DNA barcodes were only detected in genera with over three species (Supporting Information Table S5; Figure 2). In genera with three species, the species resolution rate of *rbcL* was significantly lower than that of RI, RMI, and RMPI (Supporting Information Table S5; Figure 2). Moreover, the species resolution rate of *matK* and RM were only significantly lower than that of RMI and RMPI. In genera with four species, the species resolution rate of *rbcL* was significantly lower than other barcodes except for RP. In addition, the resolution rate of RP was significantly lower than that of the barcode combinations of RI, RMI, and RMPI (Supporting Information Table S5; Figure 2). In genera with five species, however, the species resolution rate of *rbcL* was significantly lower than other barcodes except for *matK* and RP, while the differences between other barcodes were not significant (Supporting Information Table S5; Figure 2).

When we used the generalized linear models to compare species resolution rates among genera with different numbers of species, genera with four species had significantly lower rates than that of other genera by any barcode or barcode combination (Table 2; Figure 2). The species resolution rates of genera with one species were higher than that of genera with four species and three species, and only RMI and RMPI were not significant compared to genera with three species. Genera with one species were also better resolved than genera with five species, but only *rbcL*,* matK*, and RP were significant (Table 2; Figure 2). No difference was detected between genera with one species and two species, and the same pattern was found between genera with three species and five species (Table 2; Figure 2). Genera with two species were better resolved than genera with three and four species, but RI, RMI, and RMPI showed no difference in discriminating genera with two species and three species. The species resolution rates of genera with two species were also higher than that of genera with five species, but only *rbcL* and RP were significant.

**Table 2:** Differences of species discrimination with DNA barcodes between groups of genera with different numbers of species in the YFDP plot, based on a generalized linear model. Numbers in bold indicate significant differences in species discrimination. We assessed variation in percentage species discrimination for *rbcL* [R] and *matK* [M] barcodes separately, and in combination (*rbcL *+ *matK* [RM], *rbcL *+ *trnH‐psbA* [RP], *rbcL *+ ITS [RI], *rbcL *+ *matK *+ *trnH‐psbA* [RMP], *rbcL *+ *matK *+ ITS [RMI], *rbcL *+ *matK *+ *trnH‐psbA + *ITS [RMPI]. Numbers along top of table represent pairwise comparisons among genera that vary in the number of species per genus (e.g. 2 vs. 1)

		Pairwise comparison of resolution rate between genera with different numbers of species									
		2‐1	3‐1	4‐1	5‐1	3‐2	4‐2	5‐2	4‐3	5‐3	5‐4
R	*z*	2.240	10.959	16.843	8.764	8.926	15.525	7.471	8.634	0.455	7.446
	Pr(>|*z*|)	1	**<0.001**	**<0.001**	**<0.001**	**<0.001**	**<0.001**	**<0.001**	**<0.001**	1	**<0.001**
M	*z*	3.610	8.528	13.655	4.902	5.577	11.186	2.856	6.007	1.582	6.864
	Pr(>|*z*|)	0.239	**<0.001**	**<0.001**	**<0.001**	**<0.001**	**<0.001**	1	**<0.001**	1	**<0.001**
RM	*z*	1.119	8.539	13.664	3.926	7.152	12.460	3.164	6.007	2.503	7.728
	Pr(>|*z*|)	1	**<0.001**	**<0.001**	0.067	**<0.001**	**<0.001**	1	**<0.001**	1	**<0.001**
RP	*z*	1.119	7.331	14.540	4.905	6.006	13.381	4.108	8.027	0.668	7.860
	Pr(>|*z*|)	1	**<0.001**	**<0.001**	**<0.001**	**<0.001**	**<0.001**	**0.031**	**<0.001**	1	**<0.001**
RI	*z*	3.370	4.873	10.867	3.925	2.411	8.535	2.130	5.784	0.178	4.952
	Pr(>|*z*|)	0.587	**<0.001**	**<0.001**	0.068	1	**<0.001**	1	**<0.001**	1	**<0.001**
RMP	*z*	1.119	7.333	12.754	3.926	6.006	11.519	3.164	5.910	1.583	6.774
	Pr(>|*z*|)	1	**<0.001**	**<0.001**	0.067	**<0.001**	**<0.001**	1	**<0.001**	1	**<0.001**
RMI	*z*	3.372	3.662	10.869	3.926	1.285	8.535	2.130	6.664	0.997	4.952
	Pr(>|*z*|)	0.582	0.195	**<0.001**	0.067	1	**<0.001**	1	**<0.001**	1	**<0.001**
RMPI	*z*	1.119	3.662	10.869	3.926	2.607	9.611	3.164	6.664	0.997	4.952
	Pr(>|*z*|)	1	0.195	**<0.001**	0.067	1	**<0.001**	1	**<0.001**	1	**<0.001**

## 4 DISCUSSION

### 4.1 Sequence recovery

PCR and sequencing success of gymnosperms was very high with only a single pair of universal primers, and only slightly more effort was necessary for the recovery of *rbcL* and *trnH‐psbA* from angiosperms. Considerable effort, however, was required for the recovery of *matK* and *ITS* barcode regions: three pairs of primers were used for the recovery of *matK*, and even then, this region still had a relatively low sequence recovery rate. Sequence recovery was the lowest for the ITS gene region, due in large part, to fungal contaminations. Our results are comparable to other studies, which found that among the four standard plant DNA barcodes, *rbcL* was the easiest region to recover followed by *trnH‐psbA*, and other studies also encountered difficulties in the recovery of *matK* and ITS sequences (Gonzalez et al., 2009; Li et al., 2011). The sequence recovery rate in our study was higher than that of some other studies, but it is important to note that we dedicated additional effort to re‐extracting DNA and retrying PCR for DNA samples that were suspected to be contaminated with foreign plant or fungal material.

### 4.2 Species resolution

The gymnosperms in this study were all distinguished using any of the four DNA barcodes. This is not surprising given that we had six genera each with a single species in our plot, which spanned three families in the order Pinales (Supporting Information Table S1). In angiosperms, the greater sampling of multiple species per genus led to lower overall discrimination. The chloroplast gene *rbcL* resolved 80.73% of all angiosperm species in our plot (Supporting Information Table S4; Figure 1) whereas for *matK* the species resolution was 84.24%. Using two barcodes together provided slightly increased discrimination, with maximal discrimination being achieved using all four barcodes in combination (90.21%). For the two barcode combinations, RI performed best with 88.60% species resolution, marginally higher than RM (86.60%) and RP (86.01%). Adding *trnH‐psbA* and ITS region to the standard core barcode RM did not significantly increase the level of overall total species discrimination, however, the two supplemental barcodes did increase species discrimination in genera with three and four species (Figure 2).

Table 3 provides a summary of levels of species discrimination success from different floristic barcoding studies at scales that range from forest plots to regional floras. A clear signal is apparent that levels of species discrimination are lower at greater geographical scales (e.g. regional floristic barcoding projects show lower discrimination success than forest plot assessments). This is attributable to increasing the complexity of the problem (more species to discriminate among, at larger spatial scales) and increasing the likelihood of detecting intraspecific variants which may disrupt the apparent species‐level monophyly found in more geographically restricted sample sets.

**Table 3:** Comparison of species discrimination in this study with those in previous studies for forest plots and floristic regions using DNA barcodes

Study site	Area	Climate	Plants	Species/genera	Samples/species	DNA barcodes	Species resolution (%)	Reference
Yulong Mountain FDP, Yunan, China	25 ha	Subalpine temperate	Woody and herbaceous	201/135 = 1.49	491/201 = 2.44	*rbcL, matK, trnH‐psbA, ITS*	90	In this study
Wytham‐Woods, UK	18 ha	Temperate	Trees	8/8 = 1	8/8 = 1	*rbcL, matK, trnH‐psbA, ITS*	100	Pei et al. (2015)
Wabikon‐Lake, North America	25.6 ha	Temperate	Trees	10/8 = 1.25	10/10 = 1	*rbcL*,* matK*,* trnH‐psbA*	100	Pei et al. (2015)
SERC, North America	16 ha	Temperate	Trees	16/12 = 1.33	16/16 = 1	*rbcL*,* matK*,* trnH‐psbA*	93	Pei et al. (2015)
Changbaishan FDP, Jilin, China	20 ha	Temperate	Trees	14/9 = 1.56	14/14 = 1	*rbcL*,* matK*,* trnH‐psbA*	92	Pei et al. (2015)
SCBI FDP, America	25.6 ha	Temperate	Trees	35/20 = 1.75	35/35 = 1	*rbcL*,* matK*,* trnH‐psbA*	97	Pei et al. (2015)
Gutianshan FDP, Zhejiang, China	20 ha	Subtropical	Trees	91/57 = 1.60	91/91 = 1	*rbcL, matK, trnH‐psbA*	89	Pei et al. (2015)
Dinghushan FDP, Guangdong, China	20 ha	Subtropical	Trees	135/85 = 1.59	135/135 = 1	*rbcL*,* matK*,* trnH‐psbA*	91	Pei et al. (2015)
Fushan FDP, Taiwan, China	25 ha	Subtropical	Trees	69/44 = 1.57	69/69 = 1	*rbcL, matK, trnH‐psbA*	85	Pei et al. (2015)
Lienhuachih FDP, Taiwan, China	25 ha	Subtropical	Trees	96/56 = 1.71	96/96 = 1	*rbcL, matK, trnH‐psbA*	87	Pei et al. (2015)
Luquillo FDP, New Guinea	16 ha	Tropical	Trees	104/79 = 1.32	104/104 = 1	*rbcL, matK*,* trnH‐psbA*	92	Pei et al. (2015)
BCI FDP, Panama	50 ha	Tropical	Trees	296/181 = 1.64	1035/296 = 3.50	*rbcL*,* matK*,* trnH‐psbA*	98	Kress et al. (2009)
Dinghushan nature reserve, Guangdong, China	1,133 ha	Subtropical	Trees	531/260 = 2.04	971/531 = 1.83	*rbcL, matK*, ITS	94	Liu et al. (2015)
Ailaoshan nature reserve, Yunnan, China	34,483 ha	Subtropical	Trees	204/111 = 1.84	525/204 = 2.57	*rbcL*,* matK*,* trnH‐psbA*, ITS	76	Lu, Ci, Yang and Li (2013)
Xishuangbanna nature reserve, Yunnan, China	—	Tropical	Trees	655/259 = 2.53	2052/655 = 3.13	*rbcL*,* matK*,* trnH‐psbA*, ITS	60.7	Huang et al. (2015)
Ontario, Canada	348 ha	Temperate	Vascular plants	436/296 = 1.47	513/436 = 1.18	*rbcL*,* matK*,* trnH‐psbA*	95	Burgess et al. (2011)
Churchill, Canada	20,000 ha	Arctic	Vascular plants	312/147 = 2.12	900/312 = 2.88	*rbcL*,* matK*, ITS2	69	Kuzmina et al. (2012)
Canadian Arctic	—	Arctic	Vascular plants	490/178 = 2.75	2644/490 = 5.40	*rbcL*,* matK*	56	Saarela et al. (2013)
Wales, UK	2 m ha	Temperate	Seed plants	1143/455 = 2.51	4272/1143 = 3.74	*rbcL*,* matK*	74.3	De Vere et al. (2012)

Comparing directly among forest plot DNA barcoding studies, based on the same barcode combination of *rbcL* + *matK* + *trnH‐psbA*, the percentage of species discriminated is significantly negative correlated with the species‐to‐genera ratio (*R* ^2^ = 0.731, *p* < 0.001; Supporting Information Figure S2). Pei et al. (2015) also noted that species resolution rates tend to be lower with higher numbers of closely related species based on their study of DNA barcoding tree species in 13 global CTFS‐ForestGEO plots. The level of species discrimination of woody species in our plot based on *rbcL* + *matK* + *trnH‐psbA* (74.24%) is much lower than that of other plot studies (see Table 3 and Pei et al., 2015). The higher species‐to‐genera ratio of woody plants in our plot might explain the lower level of species discrimination. Compared to Pei et al. (2015) our high elevation site had a higher species‐to‐genera ratio for woody plants than 11 of their 13 plots. It is also important to note that some other studies sampled only one individual per species and based species discrimination on “non‐identical sequences between species.” This obviously can lead to an increased apparent rate of species discrimination due to an under sampling of sequence variation within a species. In our study, two to six samples were selected for each species.

### 4.3 Life form and species resolution

Despite their being twice as many herbaceous species (*N* = 129) in our plot than woody angiosperms (*N* = 66), species discrimination rates of herbaceous plants were much higher (84.92% to 97.66%) than that of woody plants (65.63%–74.24%; Supporting Information Table S4; Figure 1b). *matK* showed the greatest difference in species discrimination between these two groups of taxa, followed by barcode combinations that included the *matK* and ITS regions: differences between herbaceous plants and woody plants were much less pronounced for *rbcL* and *trnH‐psbA*. A higher species‐to‐genus ratio (SGR) for woody plants (1.65) compared to that of herbaceous plants (1.43) might be one of the reasons for lower species resolution rates among woody plants at our study site. Moreover, the slower substitution rates of woody plants results in increased time required for woody plants to accumulate sequence variation detectable by DNA barcodes (Smith & Donoghue, 2008). *Cotoneaster*,* Acer*,* Salix*,* Sorbus*, and *Rhododendron* were the major sources of resolution failures for woody species (Supporting Information Figure S1). Most of these genera are well known for hybridization and this may also contribute to low discrimination success—all of these genera showed at least some examples of highly similar sequences shared among species. For example, none of the three species of *Cotoneaster* were successfully identified in our plot. The Himalayas and the adjacent mountains of southwestern China are known to be the diversity center of this genus (Fryer & Hylmö, 2009). Widespread polyploidization and hybridization demonstrated by former studies (Fryer & Hylmö, 2009; Li et al., 2014; Li et al. 2017) help explained the inefficiency of DNA barcodes in discriminating species in this genus. DNA barcodes also did not resolve any of the four species of *Salix* in our study. It is known that *Salix* may have undergone repeated plastid capture events, widespread hybridization and dispersal and trans‐species selective sweeps that resulted in a general lack of haplotype variation for a number of standard coding and non‐coding barcode regions of the chloroplast genome (Percy et al., 2014). Similarly, the low species resolution of the four‐species genera *Acer* and *Sorbus* here may also result from the hybridization (Han et al., 2016; Li, Ohi‐Toma et al. 2017). *Rhododendron* may have undergone recent radiations and hybridization events in the mountain regions of Southwest China (Yan et al., 2015). Even though only five species coexist in our plot, and their morphological differences are clear, only one species was resolved.

Not all of the woody species showed low discrimination success. In particular, all of the species in the plot of some shrubby woody genera (e.g. *Ribes*—2 species, *Euonymus*—3 species, *Berberis*—3 species, *Lonicera*—5 species) were resolved by our DNA barcodes. For example, five species of *Lonicera* in our plot belong to distinct subsections (Hsu & Wang, 1988) with divergent relationships, thus all the species were successfully identified (inflating our discrimination success for genera with 5 species). Further sampling is required to assess whether there is a generalization here, but these data do suggest that it would be worthwhile exploring whether the association between generation time and discrimination success that is evident for herbaceous plants versus woody plants, also holds, in general, when comparing woody shrubs versus trees. The shorter time to first flowering in shrubs might be expected to lead, on average, to greater discrimination success than for more “tree‐like” woody plants whose generation time (in the sense of time to first flowering) is likely to be longer.

## 5 CONCLUSION

Our study represents one of the first applications of DNA barcoding in a subalpine forest dynamics plot. It is also one the first attempts to utilize DNA barcodes to identify both herbaceous and woody species in a plant community in the Himalya–Hengduan Mountains Region, one of the most diverse temperate floras of the world (Xing & Ree, 2017). The combination of *rbcL* and ITS, as well as the combination of *rbcL* and *matK* provided relatively high species discrimination among subalpine seed plants, although the supplemental barcodes *trnH‐psbA* and ITS increase discrimination in genera where there are more closely related species. Based on comparisons with other floristic barcoding studies, we found that level of species discrimination was strongly negatively correlated with the number of species per genus. Our study also provides a phylogenetic framework for the study of community assembly and comparative biology in this high‐elevation habitat, which has the potential to provide insights in evolutionary mechanisms underlying species coexistence in subalpine temperate forests.

## CONFLICT OF INTEREST

None declared.

## AUTHOR CONTRIBUTIONS

L.‐M.G. and D.‐Z.L. designed the study; S.‐L.T., Y.‐H.L. and K.X collected the plant samples; S.‐L.T. performed the molecular experiment; S.‐L.T. and Y.‐H.L. conducted the data analysis. S.‐L.T. and K.S. B. wrote the manuscript, P.M.H., L.‐M.G., D.‐Z.L. and Y.‐H.L. revised and edited the manuscript. All authors contributed to the final version of the text.

## DATA ACCESSIBILITY

DNA barcode sequences were deposited in GenBank (accession numbers MH116007‐MH117823, Supporting Information Table S6).
