# Supplementary material for: DNA barcoding herbaceous and woody plant species at a subalpine forest dynamics plot in Southwest China
Source: Ecol Evol. 2018 Jun 25;8(14):7195–205. doi: 10.1002/ece3.4254 (PMC6065341; doi:10.1002/ece3.4254)
Supplement: Supplementary file 1 [file ECE3-8-7195-s001.pdf]

Table S1. List of seed plants collected from the 25ha YFDP plot. Between 2013 and 2015 a total of 491 specimens representing 201 species (129 herbaceous species, 72 woody species), 135 genera of 64 families were sampled.

| Species | Family | Order | Individuals |
|---|---|---|---|
| *Acer caesium* Wall. ex Brandis | Sapindaceae | Sapindales | 4 |
| *Acer forrestii* Diels | Sapindaceae | Sapindales | 3 |
| *Acer pectinatum* Wallich ex G. Nicholson | Sapindaceae | Sapindales | 5 |
| *Acer stachyophyllum* subsp. *betulifolium* (Maxim.) P.C.DeJong | Sapindaceae | Sapindales | 3 |
| *Aconitum stapfianum* Handel-Mazzetti | Ranunculaceae | Ranunculaes | 2 |
| *Acronema astrantiifolium* H. Wolff | Apiaceae | Apiales | 2 |
| *Acronema schneideri* H.Wolff | Apiaceae | Apiales | 2 |
| *Actaea asiatica* H. Hara | Ranunculaceae | Ranunculaes | 2 |
| *Adonis davidii* Franchet | Ranunculaceae | Ranunculaes | 2 |
| *Agrostis micrantha* Steudel | Poaceae | Poales | 2 |
| *Ainsliaea lijiangensis* H.Chuang | Asteraceae | Asterales | 2 |
| *Amphicarpaea edgeworthii* Bentham in Miquel | Fabaceae | Fabales | 2 |
| *Anaphalis flaccida* Y. Ling | Asteraceae | Asterales | 4 |
| *Anaphalis likiangensis* (Franchet) Y. Ling | Asteraceae | Asterales | 2 |
| *Anaphalis margaritacea* (L.) Benth. & Hook.f. | Asteraceae | Asterales | 2 |
| *Anaphalis nepalensis* (Spreng.) Hand. -Mazz. | Asteraceae | Asterales | 2 |
| *Anemone delavayi* Franch. | Ranunculaceae | Ranunculaes | 4 |
| *Aralia chinensis* L. | Araliaceae | Apiales | 2 |
| *Arisaema auriculatum* Buchet | Araceae | Alismatales | 6 |
| *Arisaema elephas* Buchet | Araceae | Alismatales | 2 |
| *Arisaema erubescens* (Wallich) Schott in Schott & Endlicher | Araceae | Alismatales | 2 |
| *Arisaema lobatum* Engler | Araceae | Alismatales | 6 |
| *Aristolochia moupinensis* Franch. | Aristolochiaceae | Piperales | 1 |
| *Artemisia indica* Willdenow | Asteraceae | Asterales | 2 |
| *Asparagus lycopodineus* (Baker) F. T. Wang & T. Tang | Asparagaceae | Asparagales | 2 |
| *Aster albescens* var. *glabratus* (Diels) Boufford & Y. S. Chen | Asteraceae | Asterales | 2 |
| *Aster trinervius* subsp. *ageratoides* | Asteraceae | Asterales | 2 |
| *Balanophora involucrata* J. D. Hooker | Balanophoraceae | Malpiphiales | 3 |
| *Berberis fallax* C. K. Schneider | Berberidaceae | Ranunculaes | 3 |
| *Berberis papillifera* (Franch.) Koehne | Berberidaceae | Ranunculaes | 2 |
| *Berberis pruinosa* Franch. | Berberidaceae | Ranunculaes | 2 |
| *Brachypodium sylvaticum* (Hudson) P. Beauvois | Poaceae | Poales | 1 |
| *Bromus grandis* (Shear) Hitchc. | Poaceae | Poales | 2 |

| | | | |
|---|---|---|---|
| *Calamagrostis scabrescens* Griseb. | Poaceae | Poales | 1 |
| *Calanthe tricarinata* Lindley | Orchidaceae | Asparagales | 2 |
| *Cardamine hirsuta* L. | Brassicaceae | Brassicales | 2 |
| *Cardamine impatiens* L. | Brassicaceae | Brassicales | 2 |
| *Cardamine repens* (Franch.) Diels. | Brassicaceae | Brassicales | 4 |
| *Carex nubigena* D. Don ex Tilloch & Taylor | Cyperaceae | Poales | 1 |
| *Carpesium triste* Maxim. | Asteraceae | Asterales | 3 |
| *Cerastium furcatum* Chamisso & Schlechtendal | Caryophyllaceae | Carphophyllales | 2 |
| *Cerasus clarofolia* (C. K. Schneider) T. T. Yu & C. L. Li | Rosaceae | Rosales | 2 |
| *Chrysosplenium davidianum* Decaisne ex Maxim. | Saxifragaceae | Saxifragales | 2 |
| *Circaea alpina* subsp. *imaicola* (Ascherson & Magnus) Kitamura | Onagraceae | Myrtales | 2 |
| *Clematis gracilifolia* Rehder & E. H. Wilson in Sargent | Ranunculaceae | Ranunculaes | 3 |
| *Clematis montana* Buchanan-Hamilton ex de Candolle | Ranunculaceae | Ranunculaes | 4 |
| *Clinopodium repens* (Buch.-Ham. ex D. Don) Benth. | Lamiaceae | Lamiales | 6 |
| *Cornus macrophylla* rar. *macrophy* Wallich in Roxburgh | Cornaceae | Cornales | 2 |
| *Corydalis bulleyana* Diels | Papaveraceae | Ranunculaes | 2 |
| *Corydalis smithiana* Fedde | Papaveraceae | Ranunculaes | 3 |
| *Cotoneaster acutifolius* Turcz. | Rosaceae | Rosales | 2 |
| *Cotoneaster bullatus* Bois in Vilmorin & Bois | Rosaceae | Rosales | 2 |
| *Cotoneaster subadpressus* T. T. Yu | Rosaceae | Rosales | 2 |
| *Crepidium bahanense* (Handel-Mazzetti) S. C. Chen & J. J. Wood | Orchidaceae | Asparagales | 2 |
| *Cyananthus inflatus* J. D. Hooker & Thomson | Campanulaceae | Asterales | 2 |
| *Cynoglossum triste* Diels | Boraginaceae | Boraginales | 2 |
| *Cynoglossum wallichii* var. *glochidiatum* | Boraginaceae | Boraginales | 2 |
| *Daphne retusa* Hemsley | Thymelaeaceae | Malvales | 2 |
| *Delphinium thibeticum* Finet & Gagnepain | Ranunculaceae | Ranunculaes | 2 |
| *Dipelta yunnanensis* Franch. | Caprifoliaceae | Dipsacales | 3 |
| *Elatostema obtusum* Wedd. | Urticaceae | Rosales | 2 |
| *Elsholtzia ciliata* (Thunberg) Hylander | Lamiaceae | Lamiales | 2 |
| *Elsholtzia strobilifera* Bentham | Lamiaceae | Lamiales | 3 |
| *Epilobium hirsutum* Linnaeus | Onagraceae | Myrtales | 2 |
| *Euonymus lichiangensis* W.W.Sm. | Celastraceae | Celastrales | 2 |
| *Euonymus porphyreus* Loes. | Celastraceae | Celastrales | 4 |
| *Euonymus tingens* Wallich in Roxburgh | Celastraceae | Celastrales | 2 |
| *Fargesia yulongshanensis* T.P.Yi | Poaceae | Poales | 4 |
| *Festuca breviglumis* Swallen | Poaceae | Poales | 2 |

| | | | |
|---|---|---|---|
| *Festuca* sp. | Poaceae | Poales | 2 |
| *Fragaria nilgerrensis* Schlechtendal ex J. Gay | Rosaceae | Rosales | 2 |
| *Fragaria vesca* L. | Rosaceae | Rosales | 2 |
| *Galium asperifolium* Wallich | Rubiaceae | Gentianales | 2 |
| *Galium elegans* Wallich in Roxburgh | Rubiaceae | Gentianales | 3 |
| *Galium hoffmeisteri* (Klotzsch) Ehrendorfer & Schönbeck-Temesy ex R. R. Mill | Rubiaceae | Gentianales | 2 |
| *Gamblea ciliata* var. *evodiifolia* (Franchet) C. B. Shang *et al*. | Araliaceae | Apiales | 4 |
| *Gentiana maeulchanensis* Franch. | Gentianaceae | Gentianales | 1 |
| *Gentiana pubigera* C. Marquand | Gentianaceae | Gentianales | 1 |
| *Geranium delavayi* Franch. | Geraniaceae | Geraniales | 3 |
| *Geranium nepalense* Sweet | Geraniaceae | Geraniales | 4 |
| *Halenia elliptica* D. Don | Gentianaceae | Gentianales | 2 |
| *Helictotrichon delavayi* (Hackel) Henrard | Poaceae | Poales | 1 |
| *Helwingia japonica* (Thunberg) F. Dietrich | Helwingiaceae | Aquifoliales | 2 |
| *Hemiphragma heterophyllum* Wallich | Scrophulariaceae | Lamiales | 3 |
| *Hydrangea hypoglauca* Rehder | Hydrangeaceae | Cornales | 1 |
| *Hypericum bellum* H. L. Li | Hypericaceae | Malpighiales | 2 |
| *Ilex delavayi* Franch. | Aquifoliaceae | Aquifoliales | 2 |
| *Impatiens poculifer* J. D. Hooker | Balsaminaceae | Ericales | 2 |
| *Impatiens radiata* J. D. Hooker | Balsaminaceae | Ericales | 3 |
| *Isodon megathyrsus* (Diels) H. W. Li | Lamiaceae | Lamiales | 2 |
| *Juncus himalensis* Klotzsch in Klotzsch & Garcke | Juncaceae | Poales | 6 |
| *Juncus leucomelas* Royle ex D. Don | Juncaceae | Poales | 1 |
| *Ligularia hookeri* (C. B. Clarke) Hand.-Mazz. | Asteraceae | Asterales | 2 |
| *Ligusticum pteridophyllum* Franch. | Apiaceae | Apiales | 4 |
| *Ligustrum quihoui* Carriere | Oleaceae | Lamiales | 2 |
| *Litsea chunii* var. *likiangensis* Yen C. Yang & P. H. Huang | Lauraceae | Laurales | 6 |
| *Lonicera angustifolia* var. *myrtillus* (J. D. Hooker & Thomson) Q. E. Yang | Caprifoliaceae | Dipsacales | 2 |
| *Lonicera lanceolata* Wall. | Caprifoliaceae | Dipsacales | 2 |
| *Lonicera setifera* Franch. | Caprifoliaceae | Dipsacales | 3 |
| *Lonicera tangutica* Maxim. | Caprifoliaceae | Dipsacales | 3 |
| *Lonicera webbiana* Wallich ex Candolle | Caprifoliaceae | Dipsacales | 4 |
| *Maianthemum purpureum* (Wall.) LaFrankie | Asparagaceae | Asparagales | 2 |
| *Meliosma cuneifolia* Franch. | Sabiaceae | Proteales | 3 |
| *Monotropa hypopitys* L. | Ericaceae | Ericales | 1 |
| *Myriactis delavayi* Gagnep. | Asteraceae | Asterales | 3 |

| | | | |
|---|---|---|---|
| *Myriactis nepalensis* Lessing | Asteraceae | Asterales | 1 |
| *Myriactis wightii* Candolle in Wight | Asteraceae | Asterales | 2 |
| *Neottianthe oblonga* K. Y. Lang | Orchidaceae | Asparagales | 3 |
| *Ophiopogon bodinieri* H. Leveille | Asparagaceae | Asparagales | 2 |
| *Oxalis acetosella* L. | Oxalidaceae | Oxalidaceae | 2 |
| *Padus brachypoda* (Batalin) C. K. Schneider | Rosaceae | Rosales | 2 |
| *Paeonia delavayi* Franch. | Paeoniaceae | Saxifragales | 2 |
| *Panax japonicus* (T. Nees) C. A. Meyer | Araliaceae | Apiales | 6 |
| *Parasenecio latipes* (Franchet) Y. L. Chen | Asteraceae | Asterales | 3 |
| *Paris mairei* H. Leveille | Melanthiaceae | Liliales | 2 |
| *Parnassia tenella* J. D. Hooker & Thomson | Celastraceae | Celastrales | 2 |
| *Pedicularis rex* C. B. Clarke ex Maxim. | Orobanchaceae | Asparagales | 1 |
| *Philadelphus calvescens* (Rehder) S. M. Hwang | Hydrangeaceae | Cornales | 3 |
| *Phlomis ruptilis* C. Y. Wu | Lamiaceae | Lamiales | 2 |
| *Pilea auricularis* C.J. Chen | Urticaceae | Rosales | 2 |
| *Pimpinella candolleana* Wight & Arnott | Apiaceae | Apiales | 2 |
| *Plantago cavaleriei* H. Leveille | Plantaginaceae | Lamiales | 2 |
| *Pleurospermum decurrens* Franch. | Apiaceae | Apiales | 3 |
| *Poa annua* L. | Poaceae | Poales | 1 |
| *Poa acroleuca* Steudel | Poaceae | Poales | 2 |
| *Poa nemoralis* L. | Poaceae | Poales | 2 |
| *Poa trivialis* L. | Poaceae | Poales | 2 |
| *Poa* sp | Poaceae | Poales | 1 |
| *Polygonatum cirrhifolium* (Wallich) Royle | Asparagaceae | Asparagales | 2 |
| *Polygonum glaciale* (Meisner) J. D. Hooker | Polygonaceae | Caryophyllales | 2 |
| *Polygonum nepalense* Meisner | Polygonaceae | Caryophyllales | 5 |
| *Polygonum runcinatum* Buchanan-Hamilton ex D. Don | Polygonaceae | Caryophyllales | 3 |
| *Polygonum sinomontanum* Samuelsson in Hand.-Mazz. | Polygonaceae | Caryophyllales | 2 |
| *Polygonum suffultum* Maxim. | Polygonaceae | Caryophyllales | 1 |
| *Potentilla lancinata* Cardot | Rosaceae | Rosales | 2 |
| *Potentilla leuconota* D. Don | Rosaceae | Rosales | 2 |
| *Primula forrestii* I. B. Balfour in Forrest | Primulaceae | Ericales | 2 |
| *Primula septemloba* Franch. | Primulaceae | Ericales | 2 |
| *Prunella vulgaris* L. | Lamiaceae | Lamiales | 4 |
| *Pternopetalum delavayi* (Franch.) Hand-Mazz. | Apiaceae | Apiales | 2 |
| *Quercus guyavifolia* H. Leveille | Fagaceae | Fagales | 3 |
| *Rhodiola yunnanensis* (Franch.) S. H. Fu | Crassulaceae | Saxifragales | 2 |
| *Rhododendron decorum* Franch. | Ericaceae | Ericales | 3 |

| | | | |
|---|---|---|---|
| *Rhododendron rubiginosum* Franch. | Ericaceae | Ericales | 2 |
| *Rhododendron uvariifolium* Diels | Ericaceae | Ericales | 3 |
| *Rhododendron vernicosum* Franch. | Ericaceae | Ericales | 2 |
| *Rhododendron yunnanense* Franch. | Ericaceae | Ericales | 2 |
| *Ribes glaciale* Wallich in Roxburgh | Grossulariaceae | Saxifragales | 4 |
| *Ribes himalense* Royle ex Decne. | Grossulariaceae | Saxifragales | 2 |
| *Rodgersia pinnata* Franch. | Saxifragaceae | Saxifragales | 2 |
| *Rosa sericea* Lindley | Rosaceae | Rosales | 2 |
| *Rosa sertata* Rolfe | Rosaceae | Rosales | 2 |
| *Roscoea tibetica* Batalin | Zingiberaceae | Zingiberales | 2 |
| *Rubia membranacea* Diels | Rubiaceae | Gentianales | 4 |
| *Rubus fockeanus* Kurz | Rosaceae | Rosales | 2 |
| *Rubus subornatus* Focke | Rosaceae | Rosales | 3 |
| *Rumex nepalensis* Sprengel | Polygonaceae | Caryophyllales | 2 |
| *Sabia yunnanensis* Franchet. | Sabiaceae | Proteales | 4 |
| *Salix balfouriana* C.K. Schneid. | Salicaceae | Malpighiales | 1 |
| *Salix delavayana* Hand.-Mazz. | Salicaceae | Malpighiales | 3 |
| *Salix dibapha* C.K. Schneid. | Salicaceae | Malpighiales | 1 |
| *Salix eriostachya* Wall. ex Andersson | Salicaceae | Malpighiales | 1 |
| *Salvia bifidocalyx* C. Y. Wu & Y. C. Huang | Lamiaceae | Lamiales | 2 |
| *Sambucus javanica* Blume | Adoxaceae | Dipsacales | 3 |
| *Sanicula hacquetioides* Franch. | Apiaceae | Apiales | 2 |
| *Saussurrea peduncularis* Franch. | Asteraceae | Asterales | 2 |
| *Saxifraga diversifolia* Wallich ex Seringe in de Candolle | Saxifragaceae | Saxifragales | 3 |
| *Saxifraga oreophila* Franch. | Saxifragaceae | Saxifragales | 2 |
| *Saxifraga rufescens* I. B. Balfour | Saxifragaceae | Saxifragales | 2 |
| *Saxifraga strigosa* Wallich ex Seringe in de Candolle | Saxifragaceae | Saxifragales | 2 |
| *Schisandra sphaerandra* Stapf | Schisandraceae | Austrobaileyales | 2 |
| *Sedum multicaule* Wall. ex Lindl. | Crassulaceae | Saxifragales | 3 |
| *Smilax menispermoidea* A. de Candolle in A. de Candolle & C. de Candolle | Smilacaceae | Liliales | 2 |
| *Sorbus coronata* (Cardot) T.T. Yu & Tsai | Rosaceae | Rosales | 2 |
| *Sorbus hupehensis* C. K. Schneider | Rosaceae | Rosales | 2 |
| *Sorbus prattii* Koehne in Sargent | Rosaceae | Rosales | 4 |
| *Sorbus rufopilosa* C. K. Schneider | Rosaceae | Rosales | 2 |
| *Spiraea schneideriana* Rehder in Sargent | Rosaceae | Rosales | 2 |
| *Stachyurus chinensis* var. *brachystachyus* | Stachyuraceae | Crossosomatales | 2 |
| *Stellaria vestita* Kurz | Caryophyllaceae | Carphophyllales | 6 |
| *Swertia macrosperma* (C. B. Clarke) C. B. Clarke in J. D. Hooker | Gentianaceae | Gentianales | 4 |
| *Synotis erythropappa* (Bureau & Franchet) C. Jeffrey & Y. L. Chen | Asteraceae | Asterales | 3 |

| | | | |
|---|---|---|---|
| *Syringa yunnanensis* Franch. | Oleaceae | Lamiales | 2 |
| *Taraxacum dasypodum* Soest | Asteraceae | Asterales | 3 |
| *Taxillus delavayi* (Tieghem) Danser | Loranthaceae | Santalales | 3 |
| *Thalictrum delavayi* Franch. | Ranunculaceae | Ranunculaes | 3 |
| *Tilia chinensis* Maxim. | Malvaceae | Malvales | 1 |
| *Tripterospermum volubile* (D. Don) H. Hara | Gentianaceae | Gentianales | 2 |
| *Valeriana hardwickii* Wallich in Roxburgh | Caprifoliaceae | Dipsacales | 2 |
| *Veronica piroliformis* Franch. | Plantaginaceae | Lamiales | 2 |
| *Viburnum betulifolium* Batalin | Adoxaceae | Dipsacales | 3 |
| *Viola biflora* L. | Violaceae | Malpighiales | 2 |
| *Viola moupinensis* Franch. | Violaceae | Malpighiales | 2 |
| *Viola urophylla* Franch. | Violaceae | Malpighiales | 2 |
| *Youngia paleacea* (Diels) Babcock & Stebbins | Asteraceae | Asterales | 4 |
| *Juniperus squamata* Buchanan-Hamilton ex D. Don in Lambert | Cupressaceae | Pinales | 2 |
| *Abies forrestii* Coltm.-Rog. | Pinaceae | Pinales | 5 |
| *Picea likiangensis* (Franchet) E. Pritzel | Pinaceae | Pinales | 2 |
| *Pinus armandii* Franch | Pinaceae | Pinales | 2 |
| *Tsuga dumosa* (D. Don) Eichler in Engler & Prantl | Pinaceae | Pinales | 2 |
| *Taxus florinii* Spjut | Taxaceae | Pinales | 2 |

Table S2. Primers used for PCR amplification for samples of seed plant collected from the YFDP plot.

| Region | Primer Name | Sequence 5'-3' | Reference | Applications in this study |
|---|---|---|---|---|
| *rbcL* | 1F | ATGTCACCACAAACAGAAAC | Fay et al., 1997 | Seed Plants |
| | 724R | TCGCATGTACCTGCAGTAGC | Fay et al., 1997 | Seed Plants |
| *matK* | XF | TAATTTACGATCAATTCATTC | Ford et al., 2009 | Angiosperms |
| | MALPR1 | ACAAGAAAGTCGAAGTAT | Dunning & Savolainen, 2010 | Angiosperms |
| | 472F | CCCRTYCATCTGGAAATCTTGGTTC | Yu et al., 2011 | Angiosperms |
| | 1248R | GCTRTRATAATGAGAAAGATTTCTGC | Yu et al., 2011 | Angiosperms |
| | 3F_KIM | CGTACAGTACTTTTGTGTTTACGAG | Kim unpublished | Angiosperms |
| | 1R_KIM | ACCCAGTCCATCTGGAAATCTTGGTTC | Kim unpublished | Angiosperms |
| | Gym_R1A | TCAYCCGGARATTTTGGTTCG | Li et al., 2011 | Gymnosperms |
| | Gym_F1A | ATYGYRCTTTTATGTTTACARGC | Li et al., 2011 | Gymnosperms |
| *trnH-psbA* | psbAF | GTTATGCATGAACGTAATGCTC | Sang et al., 1997 | Seed Plants |
| | trnHR | CGCGCATGGTGGATTCACAAATC | Tate & Simpson, 2003 | Seed Plants |
| ITS | ITS5 | GGAAGTAAAAGTCGTAACAAGG | White et al., 1990 | Angiosperms |
| | ITS4 | TCCTCCGCTTATTGATATGC | White et al., 1990 | Angiosperms |
| ITS2 | GYM_5.8S F2 | GYAGAATCCCGTGARTCATC | Gao et al., 2012 | Gymnosperms |
| | ITS4 | TCCTCCGCTTATTGATATGC | White et al., 1990 | Gymnosperms |

Table S3. PCR and sequencing success for angiosperms and gymnosperms collected from the 25 ha YFDP plot. A total of 476 samples (195 species) of angiosperms and 15 samples (6 species) of gymnosperms were amplified and sequenced for this study.

| **Angiosperms** | **PCR** | **Sequencing** | **Gymnosperms** | **PCR** | **Sequencing** |
|---|---|---|---|---|---|
| *rbcL* | 99.37% | 98.74% | *rbcL* | 100% | 100% |
| *matK* | 93.07% | 92.02% | *matK* | 100% | 100% |
| *trnH-psbA* | 97.05% | 92.02% | *trnH-psbA* | 100% | 100% |
| ITS | 96.01% | 89.50% | ITS2 | 93.33% | 93.33% |

Table S4. Species discrimination for DNA barcode combinations for taxa collected from the YFDP plot. We assessed variation in percentage species resolution for *rbcL* [R] and *matK* [M] barcodes separately, and in combination (*rbcL* + *matK* [RM], *rbcL* + *trnH-psbA* [RP], *rbcL* + ITS [RI], *rbcL* + *matK* + *trnH-psbA* [RMP], *rbcL* + *matK* + ITS [RMI], *rbcL* + *matK* + *trnH-psbA* + ITS [RMPI].

| DNA barcodes | Genera | Species | Woody Species | Herbaceous Species |
|---|---|---|---|---|
| R | 96.06% | 80.73% | 71.21% | 84.92% |
| M | 97.56% | 84.24% | 65.63% | 92.62% |
| RM | 98.45% | 86.60% | 74.24% | 92.97% |
| RP | 96.88% | 86.01% | 72.73% | 92.91% |
| RI | 97.66% | 88.60% | 74.24% | 94.53% |
| RMP | 98.45% | 87.63% | 74.24% | 94.53% |
| RMI | 97.67% | 89.18% | 72.73% | 97.66% |
| RMPI | 98.45% | 90.21% | 74.24% | 97.66% |

Table S5. Differences of species discrimination among DNA barcodes within genera with particular number of species in the YFDP plot, based on a generalized linear model. Numbers in bold indicate significant differences in species resolution rates between the two barcodes. We assessed variation in percentage species resolution for R and M barcodes separately, and in combination RM, RP, RI, RMP, RMI, RMPI.

| Number of Species Per Genus | | 1 | 2 | 3 | 4 | 5 |
|---|---|---|---|---|---|---|
| R-M | \|z\| | 0 | 1.211 | 2.273 | 4.946 | 3.494 |
| | Pr(>\|z\|) | 1 | 1 | 1 | **<0.001** | 0.371 |
| R-RM | \|z\| | 0 | 0.877 | 2.273 | 4.946 | 4.278 |
| | Pr(>\|z\|) | 1 | 1 | 1 | **<0.001** | **0.015** |
| R-RP | \|z\| | 0 | 0.877 | 3.326 | 3.847 | 3.494 |
| | Pr(>\|z\|) | 1 | 1 | 0.686 | 0.093 | 0.371 |
| R-RI | \|z\| | 0 | 0.891 | 5.343 | 7.833 | 4.278 |
| | Pr(>\|z\|) | 1 | 1 | **<0.001** | **<0.001** | **0.015** |
| R-RMP | \|z\| | 0 | 0.877 | 3.326 | 5.972 | 4.278 |
| | Pr(>\|z\|) | 1 | 1 | 0.686 | **<0.001** | **0.015** |
| R-RMI | \|z\| | 0 | 0.891 | 6.281 | 7.833 | 4.278 |
| | Pr(>\|z\|) | 1 | 1 | **<0.001** | **<0.001** | **0.015** |
| R-RMPI | \|z\| | 0 | 0.877 | 6.281 | 7.833 | 4.278 |
| | Pr(>\|z\|) | 1 | 1 | **<0.001** | **<0.001** | **0.015** |
| M-RM | \|z\| | 0 | 2.057 | 0 | 0 | 0.803 |
| | Pr(>\|z\|) | 1 | 1 | 1 | 1 | 1 |
| M-RP | \|z\| | 0 | 2.057 | 1.061 | 1.147 | 0 |
| | Pr(>\|z\|) | 1 | 1 | 1 | 1 | 1 |
| M-RI | \|z\| | 0 | 0.349 | 3.105 | 3.119 | 0.803 |
| | Pr(>\|z\|) | 1 | 1 | 1 | 1 | 1 |
| M-RMP | \|z\| | 0 | 2.057 | 1.061 | 1.091 | 0.803 |
| | Pr(>\|z\|) | 1 | 1 | 1 | 1 | 1 |
| M-RMI | \|z\| | 0 | 0.349 | 4.061 | 3.119 | 0.803 |
| | Pr(>\|z\|) | 1 | 1 | **0.038** | 1 | 1 |
| M-RMPI | \|z\| | 0 | 2.057 | 4.061 | 3.119 | 0.803 |
| | Pr(>\|z\|) | 1 | 1 | **0.038** | 1 | 1 |
| RM-RP | \|z\| | 0 | 0 | 1.061 | 1.147 | 0.803 |
| | Pr(>\|z\|) | 1 | 1 | 1 | 1 | 1 |
| RM-RI | \|z\| | 0 | 1.767 | 3.105 | 3.119 | 0 |
| | Pr(>\|z\|) | 1 | 1 | 1 | 1 | 1 |
| RM-RMP | \|z\| | 0 | 0 | 1.061 | 1.091 | 0 |
| | Pr(>\|z\|) | 1 | 1 | 1 | 1 | 1 |
| RM-RMI | \|z\| | 0 | 1.767 | 4.061 | 3.119 | 0 |
| | Pr(>\|z\|) | 1 | 1 | **0.038** | 1 | 1 |
| RM-RMPI | \|z\| | 0 | 0 | 4.061 | 3.119 | 0 |
| | Pr(>\|z\|) | 1 | 1 | **0.038** | 1 | 1 |

| | | | | | | |
|---|---|---|---|---|---|---|
| RP-RI | \|z\| | 0 | 1.767 | 2.050 | 4.240 | 0.803 |
| | Pr(>\|z\|) | 1 | 1 | 1 | **0.017** | 1 |
| RP-RMP | \|z\| | 0 | 0 | 0 | 2.232 | 0.803 |
| | Pr(>\|z\|) | 1 | 1 | 1 | 1 | 1 |
| RP-RMI | \|z\| | 0 | 1.767 | 3.011 | 4.240 | 0.803 |
| | Pr(>\|z\|) | 1 | 1 | 1 | **0.017** | 1 |
| RP-RMPI | \|z\| | 0 | 0 | 3.011 | 4.240 | 0.803 |
| | Pr(>\|z\|) | 1 | 1 | 1 | **0.017** | 1 |
| RI-RMP | \|z\| | 0 | 1.767 | 2.050 | 2.039 | 0 |
| | Pr(>\|z\|) | 1 | 1 | 1 | 1 | 1 |
| RI-RMI | \|z\| | 0 | 0 | 0.966 | 0 | 0 |
| | Pr(>\|z\|) | 1 | 1 | 1 | 1 | 1 |
| RI-RMPI | \|z\| | 0 | 1.767 | 0.966 | 0 | 0 |
| | Pr(>\|z\|) | 1 | 1 | 1 | 1 | 1 |
| RMP-RMI | \|z\| | 0 | 1.767 | 3.011 | 2.039 | 0 |
| | Pr(>\|z\|) | 1 | 1 | 1 | 1 | 1 |
| RMP-RMPI | \|z\| | 0 | 0 | 3.011 | 2.039 | 0 |
| | Pr(>\|z\|) | 1 | 1 | 1 | 1 | 1 |
| RMI-RMPI | \|z\| | 0 | 1.767 | 0 | 0 | 0 |
| | Pr(>\|z\|) | 1 | 1 | 1 | 1 | 1 |

Table S6. GenBank accession numbers of DNA barcode sequences for the seed plants of the 25ha YFDP plot. All DNA vouchers were deposited in the Herbarium of Kunming Institute of Botany (KUN), Chinese Academy of Sciences.

| **Species** | **Vouchers** | ***rbcL*** **GenBank accession No.** | ***matK*** **GenBank accession No.** | ***trnH-psbA*** **GenBank accession No.** | **ITS GenBank accession No.** |
|---|---|---|---|---|---|
| *Acer caesium* | YLDP002A | MH116007 | MH116489 | MH116939 | MH117387 |
| *Acer caesium* | YLDP002B | MH116008 | MH116490 | MH116940 | MH117388 |
| *Acer caesium* | YLDP002C | MH116009 | — | MH116941 | MH117389 |
| *Acer caesium* | YLDP002D | MH116010 | MH116491 | MH116942 | MH117390 |
| *Acer forrestii* | YLDP143A | MH116011 | MH116492 | — | MH117391 |
| *Acer forrestii* | YLDP171A | MH116012 | MH116493 | MH116943 | MH117392 |
| *Acer forrestii* | YLDP171B | MH116013 | MH116494 | MH116944 | MH117393 |
| *Acer pectinatum* | YLDP004A | MH116014 | MH116495 | MH116945 | MH117394 |
| *Acer pectinatum* | YLDP004B | MH116015 | MH116496 | MH116946 | MH117395 |
| *Acer pectinatum* | YLDP082A | MH116016 | MH116497 | MH116947 | MH117396 |
| *Acer pectinatum* | YLDP082D | MH116017 | MH116498 | MH116948 | MH117397 |
| *Acer pectinatum* | YLDP288A | MH116018 | MH116499 | MH116949 | MH117398 |
| *Acer stachyophyllum* subsp. *betulifolium* | YLDP084A | MH116019 | MH116500 | MH116950 | MH117399 |
| *Acer stachyophyllum* subsp. *betulifolium* | YLDP084B | MH116020 | MH116501 | MH116951 | MH117400 |
| *Acer stachyophyllum* subsp. *betulifolium* | YLDP149A | MH116021 | MH116502 | MH116952 | MH117401 |
| *Aconitum stapfianum* | YLDP209A | MH116022 | MH116503 | MH116953 | MH117402 |
| *Aconitum stapfianum* | YLDP209B | MH116023 | MH116504 | MH116954 | MH117403 |
| *Acronema astrantiifolium* | YLDP227A | MH116024 | MH116505 | MH116955 | MH117404 |
| *Acronema astrantiifolium* | YLDP227C | MH116025 | MH116506 | MH116956 | — |
| *Acronema schneideri* | YLDP244A | MH116026 | MH116507 | MH116957 | MH117405 |
| *Acronema schneideri* | YLDP244B | MH116027 | MH116508 | MH116958 | MH117406 |
| *Actaea cimicifuga* | YLDP136A | MH116028 | MH116509 | MH116959 | MH117407 |
| *Actaea cimicifuga* | YLDP136B | MH116029 | MH116510 | MH116960 | MH117408 |
| *Adonis davidii* | YLDP113A | MH116030 | MH116511 | MH116961 | MH117409 |
| *Adonis davidii* | YLDP199A | MH116031 | MH116512 | MH116962 | MH117410 |
| *Agrostis micrantha* | YLDP174A | MH116032 | MH116513 | MH116963 | MH117411 |
| *Agrostis micrantha* | YLDP174B | MH116033 | MH116514 | MH116964 | MH117412 |
| *Ainsliaea lijiangensis* | YLDP219A | MH116034 | MH116515 | MH116965 | MH117413 |
| *Ainsliaea lijiangensis* | YLDP219E | MH116035 | MH116516 | MH116966 | MH117414 |
| *Amphicarpaea edgeworthii* | YLDP211A | — | MH116517 | — | MH117415 |

| | | | | | |
|---|---|---|---|---|---|
| *Amphicarpaea edgeworthii* | YLDP211B | MH116036 | MH116518 | MH116967 | MH117416 |
| *Anaphalis flaccida* | YLDP216A | MH116037 | MH116519 | MH116968 | MH117417 |
| *Anaphalis flaccida* | YLDP216C | MH116038 | MH116520 | MH116969 | MH117418 |
| *Anaphalis flaccida* | YLDP252A | MH116039 | MH116521 | MH116970 | MH117419 |
| *Anaphalis flaccida* | YLDP252B | MH116040 | MH116522 | MH116971 | MH117420 |
| *Anaphalis likiangensis* | YLDP225A | MH116041 | MH116523 | MH116972 | MH117421 |
| *Anaphalis likiangensis* | YLDP225B | MH116042 | MH116524 | MH116973 | MH117422 |
| *Anaphalis margaritacea* | YLDP258A | MH116043 | MH116525 | MH116974 | MH117423 |
| *Anaphalis margaritacea* | YLDP258B | MH116044 | MH116526 | MH116975 | MH117424 |
| *Anaphalis nepalensis* | YLDP259A | MH116045 | MH116527 | MH116976 | MH117425 |
| *Anaphalis nepalensis* | YLDP259B | MH116046 | MH116528 | MH116977 | MH117426 |
| *Anemone delavayi* | YLDP091A | MH116047 | MH116529 | MH116978 | MH117427 |
| *Anemone delavayi* | YLDP091B | MH116048 | MH116530 | MH116979 | MH117428 |
| *Anemone delavayi* | YLDP124A | MH116049 | MH116531 | MH116980 | MH117429 |
| *Anemone delavayi* | YLDP124C | MH116050 | MH116532 | MH116981 | MH117430 |
| *Aralia chinensis* | YLDP038A | MH116051 | MH116533 | MH116982 | MH117431 |
| *Aralia chinensis* | YLDP114A | MH116052 | MH116534 | MH116983 | MH117432 |
| *Arisaema auriculatum* | YLDP073A | MH116053 | MH116535 | — | MH117433 |
| *Arisaema auriculatum* | YLDP073B | MH116054 | MH116536 | — | MH117434 |
| *Arisaema auriculatum* | YLDP116A | MH116055 | MH116537 | — | MH117435 |
| *Arisaema auriculatum* | YLDP118A | MH116056 | MH116538 | — | MH117436 |
| *Arisaema auriculatum* | YLDP166A | MH116057 | MH116539 | MH116984 | MH117437 |
| *Arisaema auriculatum* | YLDP166B | MH116058 | MH116540 | MH116985 | MH117438 |
| *Arisaema elephas* | YLDP087A | MH116059 | MH116541 | MH116986 | MH117439 |
| *Arisaema elephas* | YLDP087B | MH116060 | MH116542 | MH116987 | MH117440 |
| *Arisaema erubescens* | YLDP122A | MH116061 | MH116543 | MH116988 | MH117441 |
| *Arisaema erubescens* | YLDP122B | MH116062 | MH116544 | MH116989 | MH117442 |
| *Arisaema lobatum* | YLDP098A | MH116063 | MH116545 | MH116990 | MH117443 |
| *Arisaema lobatum* | YLDP148A | MH116064 | MH116546 | MH116991 | MH117444 |
| *Arisaema lobatum* | YLDP162A | MH116065 | MH116547 | — | MH117445 |
| *Arisaema lobatum* | YLDP162B | MH116066 | MH116548 | MH116992 | MH117446 |
| *Arisaema lobatum* | YLDP164A | MH116067 | MH116549 | MH116993 | MH117447 |
| *Arisaema lobatum* | YLDP164B | MH116068 | MH116550 | MH116994 | MH117448 |
| *Aristolochia moupinensis* | YLDP303A | MH116069 | MH116551 | MH116995 | — |
| *Artemisia indica* | YLDP294A | MH116070 | MH116552 | MH116996 | MH117449 |
| *Artemisia indica* | YLDP294C | MH116071 | MH116553 | MH116997 | MH117450 |
| *Asparagus lycopodineus* | YLDP248A | MH116072 | MH116554 | MH116998 | MH117451 |

| | | | | | |
|---|---|---|---|---|---|
| *Asparagus lycopodineus* | YLDP248C | MH116073 | MH116555 | MH116999 | MH117452 |
| *Aster albescens* var. *glabratus* | YLDP229A | MH116074 | MH116556 | MH117000 | MH117453 |
| *Aster albescens* var. *glabratus* | YLDP229B | MH116075 | MH116557 | MH117001 | MH117454 |
| *Aster trinervius* subsp. *ageratoides* | YLDP237A | MH116076 | MH116558 | MH117002 | MH117455 |
| *Aster trinervius* subsp. *ageratoides* | YLDP237B | MH116077 | MH116559 | MH117003 | — |
| *Berberis fallax* | YLDP016A | MH116078 | MH116560 | MH117004 | MH117456 |
| *Berberis fallax* | YLDP016B | MH116079 | MH116561 | MH117005 | MH117457 |
| *Berberis fallax* | YLDP016C | MH116080 | MH116562 | MH117006 | MH117458 |
| *Berberis papillifera* | YLDP068B | MH116081 | — | — | MH117459 |
| *Berberis papillifera* | YLDP068C | MH116082 | — | MH117007 | MH117460 |
| *Berberis pruinosa* | YLDP096C | MH116083 | — | MH117008 | MH117461 |
| *Berberis pruinosa* | YLDP096D | MH116084 | MH116563 | — | — |
| *Brachypodium sylvaticum* | YLDP175A | MH116085 | — | MH117009 | MH117462 |
| *Bromus grandis* | YLDP228A | MH116086 | MH116564 | MH117010 | MH117463 |
| *Bromus grandis* | YLDP228B | MH116087 | MH116565 | MH117011 | MH117464 |
| *Calamagrostis scabrescens* | YLDP290A | MH116088 | MH116566 | MH117012 | — |
| *Calanthe tricarinata* | YLDP159A | MH116089 | MH116567 | MH117013 | MH117465 |
| *Calanthe tricarinata* | YLDP159B | MH116090 | MH116568 | MH117014 | MH117466 |
| *Cardamine hirsuta* | YLDP123A | MH116091 | MH116569 | MH117015 | MH117467 |
| *Cardamine hirsuta* | YLDP123B | MH116092 | MH116570 | MH117016 | MH117468 |
| *Cardamine impatiens* | YLDP163A | MH116093 | MH116571 | MH117017 | MH117469 |
| *Cardamine impatiens* | YLDP163B | MH116094 | MH116572 | MH117018 | MH117470 |
| *Cardamine repens* | YLDP105A | MH116095 | MH116573 | MH117019 | MH117471 |
| *Cardamine repens* | YLDP105B | MH116096 | MH116574 | MH117020 | MH117472 |
| *Cardamine repens* | YLDP165A | MH116097 | MH116575 | MH117021 | MH117473 |
| *Cardamine repens* | YLDP165B | MH116098 | MH116576 | MH117022 | MH117474 |
| *Carex nubigena* | YLDP266A | MH116099 | — | — | MH117475 |
| *Carpesium triste* | YLDP045A | MH116100 | MH116577 | MH117023 | MH117476 |
| *Carpesium triste* | YLDP045B | MH116101 | — | MH117024 | MH117477 |
| *Carpesium triste* | YLDP045C | MH116102 | — | MH117025 | MH117478 |
| *Cerastium furcatum* | YLDP151A | MH116103 | MH116578 | MH117026 | MH117479 |
| *Cerastium furcatum* | YLDP151B | MH116104 | — | MH117027 | MH117480 |
| *Cerasus clarofolia* | YLDP025A | MH116105 | MH116579 | MH117028 | MH117481 |
| *Cerasus clarofolia* | YLDP025B | MH116106 | MH116580 | MH117029 | MH117482 |
| *Chrysosplenium davidianum* | YLDP070A | MH116107 | MH116581 | MH117030 | — |
| *Chrysosplenium davidianum* | YLDP070C | MH116108 | MH116582 | MH117031 | — |
| *Circaea alpina* subsp. *imaicola* | YLDP022A | MH116109 | MH116583 | MH117032 | — |

| | | | | | |
|---|---|---|---|---|---|
| *Circaea alpina* subsp. *imaicola* | YLDP022B | MH116110 | MH116584 | MH117033 | MH117483 |
| *Clematis gracilifolia* | YLDP085A | MH116111 | MH116585 | MH117034 | MH117484 |
| *Clematis gracilifolia* | YLDP085B | MH116112 | MH116586 | MH117035 | MH117485 |
| *Clematis gracilifolia* | YLDP086A | MH116113 | MH116587 | — | MH117486 |
| *Clematis montana* | YLDP039A | MH116114 | MH116588 | MH117036 | MH117487 |
| *Clematis montana* | YLDP039B | MH116115 | MH116589 | MH117037 | MH117488 |
| *Clematis montana* | YLDP039C | MH116116 | MH116590 | MH117038 | MH117489 |
| *Clematis montana* | YLDP106A | MH116117 | MH116591 | MH117039 | MH117490 |
| *Clinopodium repens* | YLDP053A | MH116118 | MH116592 | MH117040 | — |
| *Clinopodium repens* | YLDP053B | MH116119 | MH116593 | MH117041 | — |
| *Clinopodium repens* | YLDP053E | MH116120 | MH116594 | MH117042 | — |
| *Clinopodium repens* | YLDP053F | MH116121 | MH116595 | MH117043 | — |
| *Clinopodium repens* | YLDP130A | MH116122 | MH116596 | MH117044 | — |
| *Clinopodium repens* | YLDP130B | MH116123 | MH116597 | MH117045 | — |
| *Cornus macrophylla* var. *macrophy* | YLDP144A | MH116124 | MH116598 | MH117046 | — |
| *Cornus macrophylla* var. *macrophy* | YLDP144B | MH116125 | MH116599 | MH117047 | MH117491 |
| *Corydalis bulleyana* | YLDP089A | MH116126 | MH116600 | MH117048 | MH117492 |
| *Corydalis bulleyana* | YLDP089B | MH116127 | MH116601 | MH117049 | MH117493 |
| *Corydalis smithiana* | YLDP153A | MH116128 | MH116602 | MH117050 | MH117494 |
| *Corydalis smithiana* | YLDP153B | MH116129 | MH116603 | MH117051 | MH117495 |
| *Corydalis smithiana* | YLDP161A | MH116130 | MH116604 | MH117052 | MH117496 |
| *Cotoneaster acutifolius* | YLDP110A | MH116131 | MH116605 | — | MH117497 |
| *Cotoneaster acutifolius* | YLDP110B | MH116132 | MH116606 | MH117053 | — |
| *Cotoneaster bullatus* | YLDP152A | MH116133 | MH116607 | — | — |
| *Cotoneaster bullatus* | YLDP152B | MH116134 | MH116608 | — | — |
| *Cotoneaster subadpressus* | YLDP236A | MH116135 | MH116609 | MH117054 | MH117498 |
| *Cotoneaster subadpressus* | YLDP236B | MH116136 | MH116610 | MH117055 | MH117499 |
| *Crepidium bahanense* | YLDP125A | MH116137 | MH116611 | MH117056 | MH117500 |
| *Crepidium bahanense* | YLDP125B | MH116138 | MH116612 | MH117057 | MH117501 |
| *Cyananthus inflatus* | YLDP261B | MH116139 | MH116613 | MH117058 | MH117502 |
| *Cyananthus inflatus* | YLDP261C | MH116140 | MH116614 | MH117059 | MH117503 |
| *Cynoglossum triste* | YLDP065A | MH116141 | MH116615 | MH117060 | MH117504 |
| *Cynoglossum triste* | YLDP076A | MH116142 | MH116616 | MH117061 | MH117505 |
| *Cynoglossum wallichii* var. *glochidiatum* | YLDP168A | MH116143 | MH116617 | MH117062 | MH117506 |
| *Cynoglossum wallichii* var. *glochidiatum* | YLDP168B | MH116144 | MH116618 | MH117063 | MH117507 |
| *Daphne retusa* | YLDP107A | MH116145 | MH116619 | — | MH117508 |
| *Daphne retusa* | YLDP107B | MH116146 | MH116620 | — | MH117509 |

| | | | | | |
|---|---|---|---|---|---|
| *Delphinium thibeticum* | YLDP221A | MH116147 | MH116621 | MH117064 | MH117510 |
| *Delphinium thibeticum* | YLDP221B | MH116148 | MH116622 | MH117065 | MH117511 |
| *Dipelta yunnanensis* | YLDP060A | MH116149 | MH116623 | MH117066 | MH117512 |
| *Dipelta yunnanensis* | YLDP060B | MH116150 | MH116624 | MH117067 | MH117513 |
| *Dipelta yunnanensis* | YLDP060C | MH116151 | MH116625 | MH117068 | MH117514 |
| *Elatostema obtusum* | YLDP090A | MH116152 | MH116626 | MH117069 | MH117515 |
| *Elatostema obtusum* | YLDP090B | MH116153 | MH116627 | MH117070 | MH117516 |
| *Elsholtzia ciliata* | YLDP041A | MH116154 | MH116628 | MH117071 | MH117517 |
| *Elsholtzia ciliata* | YLDP041B | MH116155 | MH116629 | MH117072 | MH117518 |
| *Elsholtzia strobilifera* | YLDP044A | MH116156 | MH116630 | MH117073 | MH117519 |
| *Elsholtzia strobilifera* | YLDP044C | MH116157 | MH116631 | MH117074 | MH117520 |
| *Elsholtzia strobilifera* | YLDP044D | MH116158 | MH116632 | MH117075 | MH117521 |
| *Epilobium hirsutum* | YLDP049A | MH116159 | MH116633 | — | MH117522 |
| *Epilobium hirsutum* | YLDP049B | MH116160 | MH116634 | — | MH117523 |
| *Euonymus lichiangensis* | YLDP243A | MH116161 | MH116635 | MH117076 | MH117524 |
| *Euonymus lichiangensis* | YLDP243B | MH116162 | MH116636 | MH117077 | MH117525 |
| *Euonymus porphyreus* | YLDP017A | MH116163 | MH116637 | MH117078 | MH117526 |
| *Euonymus porphyreus* | YLDP017B | MH116164 | MH116638 | MH117079 | MH117527 |
| *Euonymus porphyreus* | YLDP095A | MH116165 | MH116639 | MH117080 | MH117528 |
| *Euonymus porphyreus* | YLDP095B | MH116166 | MH116640 | MH117081 | MH117529 |
| *Euonymus tingens* | YLDP064A | MH116167 | MH116641 | MH117082 | MH117530 |
| *Euonymus tingens* | YLDP064B | MH116168 | MH116642 | MH117083 | MH117531 |
| *Fargesia yulongshanensis* | YLDP013A | MH116169 | MH116643 | MH117084 | MH117532 |
| *Fargesia yulongshanensis* | YLDP013B | MH116170 | MH116644 | MH117085 | MH117533 |
| *Fargesia yulongshanensis* | YLDP024A | MH116171 | MH116645 | MH117086 | — |
| *Fargesia yulongshanensis* | YLDP024B | MH116172 | MH116646 | MH117087 | — |
| *Festuca breviglumis* | YLDP189A | MH116173 | MH116647 | MH117088 | MH117534 |
| *Festuca breviglumis* | YLDP189B | MH116174 | MH116648 | MH117089 | MH117535 |
| *Festuca sp* | YLDP282A | MH116175 | MH116649 | MH117090 | MH117536 |
| *Festuca sp* | YLDP283A | MH116176 | — | MH117091 | MH117537 |
| *Fragaria nilgerrensis* | YLDP112A | MH116177 | MH116650 | MH117092 | MH117538 |
| *Fragaria nilgerrensis* | YLDP112B | MH116178 | MH116651 | MH117093 | MH117539 |
| *Fragaria vesca* | YLDP088A | MH116179 | MH116652 | MH117094 | MH117540 |
| *Fragaria vesca* | YLDP088B | MH116180 | MH116653 | MH117095 | MH117541 |
| *Galium asperifolium* | YLDP251A | MH116181 | MH116654 | MH117096 | MH117542 |
| *Galium asperifolium* | YLDP251B | MH116182 | MH116655 | MH117097 | MH117543 |
| *Galium elegans* | YLDP050A | MH116183 | MH116656 | MH117098 | MH117544 |

| | | | | | |
|---|---|---|---|---|---|
| *Galium elegans* | YLDP050B | MH116184 | MH116657 | MH117099 | MH117545 |
| *Galium elegans* | YLDP050C | MH116185 | MH116658 | MH117100 | MH117546 |
| *Galium hoffmeisteri* | YLDP126A | MH116186 | — | MH117101 | MH117547 |
| *Galium hoffmeisteri* | YLDP126B | MH116187 | — | MH117102 | MH117548 |
| *Gamblea ciliata* var. *evodiifolia* | YLDP012A | MH116188 | MH116659 | MH117103 | MH117549 |
| *Gamblea ciliata* var. *evodiifolia* | YLDP012B | MH116189 | MH116660 | MH117104 | MH117550 |
| *Gamblea ciliata* var. *evodiifolia* | YLDP083A | MH116190 | MH116661 | MH117105 | MH117551 |
| *Gamblea ciliata* var. *evodiifolia* | YLDP083B | MH116191 | MH116662 | MH117106 | MH117552 |
| *Gentiana maeulchanensis* | YLDP100B | MH116192 | MH116663 | MH117107 | MH117553 |
| *Gentiana pubigera* | YLDP100A | MH116193 | MH116664 | MH117108 | MH117554 |
| *Geranium delavayi* | YLDP056A | MH116194 | — | MH117109 | MH117555 |
| *Geranium delavayi* | YLDP056B | MH116195 | — | MH117110 | MH117556 |
| *Geranium delavayi* | YLDP056D | MH116196 | — | MH117111 | MH117557 |
| *Geranium nepalense* | YLDP055A | MH116197 | — | MH117112 | MH117558 |
| *Geranium nepalense* | YLDP055B | MH116198 | — | MH117113 | MH117559 |
| *Geranium nepalense* | YLDP160A | MH116199 | — | MH117114 | MH117560 |
| *Geranium nepalense* | YLDP160B | MH116200 | — | MH117115 | MH117561 |
| *Halenia elliptica* | YLDP214B | MH116201 | MH116665 | MH117116 | MH117562 |
| *Halenia elliptica* | YLDP214C | MH116202 | MH116666 | MH117117 | MH117563 |
| *Helictotrichon delavayi* | YLDP111A | MH116203 | MH116667 | MH117118 | MH117564 |
| *Helwingia japonica* | YLDP270C | MH116204 | MH116668 | MH117119 | MH117565 |
| *Helwingia japonica* | YLDP270D | MH116205 | MH116669 | MH117120 | MH117566 |
| *Hemiphragma heterophyllum* | YLDP042A | MH116206 | MH116670 | MH117121 | MH117567 |
| *Hemiphragma heterophyllum* | YLDP042B | MH116207 | MH116671 | MH117122 | MH117568 |
| *Hemiphragma heterophyllum* | YLDP042C | MH116208 | MH116672 | MH117123 | MH117569 |
| *Hydrangea hypoglauca* | YLDP195A | MH116209 | MH116673 | MH117124 | MH117570 |
| *Hypericum bellum* | YLDP190A | MH116210 | — | MH117125 | MH117571 |
| *Hypericum bellum* | YLDP190B | MH116211 | — | MH117126 | MH117572 |
| *Ilex delavayi* | YLDP020A | MH116212 | MH116674 | MH117127 | MH117573 |
| *Ilex delavayi* | YLDP020B | MH116213 | MH116675 | MH117128 | MH117574 |
| *Impatiens poculifer* | YLDP015A | MH116214 | MH116676 | MH117129 | MH117575 |
| *Impatiens poculifer* | YLDP015B | MH116215 | MH116677 | MH117130 | MH117576 |
| *Impatiens radiata* | YLDP031A | MH116216 | MH116678 | MH117131 | MH117577 |
| *Impatiens radiata* | YLDP031B | MH116217 | MH116679 | MH117132 | MH117578 |
| *Impatiens radiata* | YLDP031C | MH116218 | MH116680 | MH117133 | MH117579 |
| *Isodon megathyrsus* | YLDP037A | MH116219 | MH116681 | MH117134 | MH117580 |
| *Isodon megathyrsus* | YLDP037B | MH116220 | MH116682 | MH117135 | MH117581 |

| | | | | | |
|---|---|---|---|---|---|
| *Juncus himalensis* | YLDP178B | MH116221 | MH116683 | — | MH117582 |
| *Juncus himalensis* | YLDP191B | MH116222 | MH116684 | — | MH117583 |
| *Juncus himalensis* | YLDP191C | MH116223 | MH116685 | — | MH117584 |
| *Juncus himalensis* | YLDP206A | MH116224 | MH116686 | — | MH117585 |
| *Juncus himalensis* | YLDP206B | MH116225 | MH116687 | — | MH117586 |
| *Juncus himalensis* | YLDP267A | MH116226 | — | — | MH117587 |
| *Juncus leucomelas* | YLDP178A | MH116227 | — | — | MH117588 |
| *Ligularia hookeri* | YLDP246A | MH116228 | MH116688 | MH117136 | MH117589 |
| *Ligularia hookeri* | YLDP246B | MH116229 | MH116689 | MH117137 | MH117590 |
| *Ligusticum pteridophyllum* | YLDP051A | MH116230 | MH116690 | MH117138 | MH117591 |
| *Ligusticum pteridophyllum* | YLDP051B | MH116231 | MH116691 | MH117139 | MH117592 |
| *Ligusticum pteridophyllum* | YLDP250A | MH116232 | MH116692 | MH117140 | MH117593 |
| *Ligusticum pteridophyllum* | YLDP250B | MH116233 | MH116693 | MH117141 | MH117594 |
| *Ligustrum quihoui* | YLDP272A | MH116234 | MH116694 | MH117142 | — |
| *Ligustrum quihoui* | YLDP272B | MH116235 | MH116695 | MH117143 | MH117595 |
| *Litsea chunii* var. *likiangensis* | YLDP009A | MH116236 | MH116696 | MH117144 | — |
| *Litsea chunii* var. *likiangensis* | YLDP009B | MH116237 | MH116697 | MH117145 | — |
| *Litsea chunii* var. *likiangensis* | YLDP077A | MH116238 | MH116698 | MH117146 | — |
| *Litsea chunii* var. *likiangensis* | YLDP145A | MH116239 | MH116699 | MH117147 | — |
| *Litsea chunii* var. *likiangensis* | YLDP145B | MH116240 | MH116700 | MH117148 | MH117596 |
| *Litsea chunii* var. *likiangensis* | YLDP275A | MH116241 | MH116701 | MH117149 | — |
| *Lonicera angustifolia* var. *myrtillus* | YLDP108A | MH116242 | MH116702 | MH117150 | MH117597 |
| *Lonicera angustifolia* var. *myrtillus* | YLDP108C | MH116243 | — | MH117151 | MH117598 |
| *Lonicera lanceolata* | YLDP139A | MH116244 | MH116703 | MH117152 | MH117599 |
| *Lonicera lanceolata* | YLDP139C | MH116245 | MH116704 | MH117153 | MH117600 |
| *Lonicera setifera* | YLDP029A | MH116246 | MH116705 | MH117154 | — |
| *Lonicera setifera* | YLDP029B | MH116247 | MH116706 | MH117155 | — |
| *Lonicera setifera* | YLDP029C | MH116248 | MH116707 | MH117156 | MH117601 |
| *Lonicera tangutica* | YLDP001A | MH116249 | MH116708 | MH117157 | — |
| *Lonicera tangutica* | YLDP001B | MH116250 | MH116709 | MH117158 | — |
| *Lonicera tangutica* | YLDP001C | MH116251 | MH116710 | MH117159 | MH117602 |
| *Lonicera webbiana* | YLDP007A | MH116252 | MH116711 | MH117160 | MH117603 |
| *Lonicera webbiana* | YLDP007B | MH116253 | MH116712 | MH117161 | MH117604 |
| *Lonicera webbiana* | YLDP007C | MH116254 | MH116713 | MH117162 | MH117605 |
| *Lonicera webbiana* | YLDP007D | MH116255 | MH116714 | MH117163 | MH117606 |
| *Maianthemum purpureum* | YLDP131A | MH116256 | MH116715 | MH117164 | MH117607 |
| *Maianthemum purpureum* | YLDP131B | MH116257 | MH116716 | MH117165 | — |

| | | | | | |
|---|---|---|---|---|---|
| *Meliosma cuneifolia* | YLDP008A | MH116258 | — | MH117166 | MH117608 |
| *Meliosma cuneifolia* | YLDP008B | MH116259 | — | MH117167 | MH117609 |
| *Meliosma cuneifolia* | YLDP008C | MH116260 | — | MH117168 | MH117610 |
| *Monotropa hypopitys* | YLDP305A | — | MH116717 | — | — |
| *Myriactis delavayi* | YLDP028A | MH116261 | MH116718 | MH117169 | MH117611 |
| *Myriactis delavayi* | YLDP028B | MH116262 | MH116719 | MH117170 | MH117612 |
| *Myriactis delavayi* | YLDP028C | MH116263 | MH116720 | MH117171 | MH117613 |
| *Myriactis nepalensis* | YLDP299A | MH116264 | MH116721 | MH117172 | MH117614 |
| *Myriactis wightii* | YLDP027A | MH116265 | MH116722 | MH117173 | MH117615 |
| *Myriactis wightii* | YLDP027B | MH116266 | MH116723 | MH117174 | MH117616 |
| *Neottianthe oblonga* | YLDP218A | MH116267 | MH116724 | MH117175 | MH117617 |
| *Neottianthe oblonga* | YLDP218B | MH116268 | MH116725 | MH117176 | MH117618 |
| *Neottianthe oblonga* | YLDP218D | MH116269 | MH116726 | MH117177 | MH117619 |
| *Ophiopogon bodinieri* | YLDP134A | MH116270 | MH116727 | MH117178 | MH117620 |
| *Ophiopogon bodinieri* | YLDP134B | MH116271 | MH116728 | MH117179 | MH117621 |
| *Oxalis acetosella* | YLDP069A | MH116272 | MH116729 | MH117180 | MH117622 |
| *Oxalis acetosella* | YLDP069C | MH116273 | MH116730 | MH117181 | MH117623 |
| *Padus brachypoda* | YLDP081A | MH116274 | MH116731 | MH117182 | MH117624 |
| *Padus brachypoda* | YLDP081B | MH116275 | MH116732 | MH117183 | MH117625 |
| *Paeonia delavayi* | YLDP066A | MH116276 | MH116733 | MH117184 | MH117626 |
| *Paeonia delavayi* | YLDP066B | MH116277 | MH116734 | MH117185 | MH117627 |
| *Panax japonicus* var. *bipinnatifidus* | YLDP203A | MH116278 | MH116735 | MH117186 | MH117628 |
| *Panax japonicus* var. *bipinnatifidus* | YLDP203B | MH116279 | MH116736 | MH117187 | MH117629 |
| *Panax japonicus* | YLDP128A | MH116280 | MH116737 | MH117188 | MH117630 |
| *Panax japonicus* | YLDP128B | MH116281 | MH116738 | MH117189 | MH117631 |
| *Panax japonicus* | YLDP176A | MH116282 | MH116739 | MH117190 | MH117632 |
| *Panax japonicus* | YLDP176B | MH116283 | MH116740 | MH117191 | MH117633 |
| *Parasenecio latipes* | YLDP034A | MH116284 | MH116741 | MH117192 | MH117634 |
| *Parasenecio latipes* | YLDP034B | MH116285 | MH116742 | MH117193 | MH117635 |
| *Parasenecio latipes* | YLDP034C | MH116286 | MH116743 | MH117194 | MH117636 |
| *Paris mairei* | YLDP115A | MH116287 | — | MH117195 | MH117637 |
| *Paris mairei* | YLDP115C | MH116288 | — | MH117196 | MH117638 |
| *Parnassia tenella* | YLDP262A | MH116289 | — | MH117197 | MH117639 |
| *Parnassia tenella* | YLDP262B | MH116290 | — | MH117198 | MH117640 |
| *Pedicularis rex* | YLDP253A | MH116291 | MH116744 | MH117199 | MH117641 |
| *Philadelphus calvescens* | YLDP010A | MH116292 | MH116745 | MH117200 | MH117642 |
| *Philadelphus calvescens* | YLDP010B | MH116293 | MH116746 | MH117201 | MH117643 |

| | | | | | |
|---|---|---|---|---|---|
| *Philadelphus calvescens* | YLDP010D | MH116294 | MH116747 | MH117202 | MH117644 |
| *Phlomis ruptilis* | YLDP233A | MH116295 | MH116748 | MH117203 | — |
| *Phlomis ruptilis* | YLDP233C | MH116296 | MH116749 | MH117204 | MH117645 |
| *Pilea auricularis* | YLDP033A | MH116297 | MH116750 | MH117205 | MH117646 |
| *Pilea auricularis* | YLDP033B | MH116298 | MH116751 | MH117206 | MH117647 |
| *Pimpinella candolleana* | YLDP245A | MH116299 | MH116752 | MH117207 | MH117648 |
| *Pimpinella candolleana* | YLDP245B | MH116300 | MH116753 | — | MH117649 |
| *Plantago cavaleriei* | YLDP150A | MH116301 | MH116754 | MH117208 | MH117650 |
| *Plantago cavaleriei* | YLDP150B | MH116302 | MH116755 | MH117209 | MH117651 |
| *Pleurospermum decurrens* | YLDP199C | MH116303 | MH116756 | MH117210 | MH117652 |
| *Pleurospermum decurrens* | YLDP199F | MH116304 | MH116757 | MH117211 | MH117653 |
| *Pleurospermum decurrens* | YLDP199G | MH116305 | MH116758 | MH117212 | MH117654 |
| *Poa acroleuca* | YLDP146A | MH116306 | MH116759 | MH117213 | MH117655 |
| *Poa acroleuca* | YLDP154B | MH116307 | MH116760 | MH117214 | MH117656 |
| *Poa annua* | YLDP104A | MH116308 | MH116761 | MH117215 | MH117657 |
| *Poa nemoralis* | YLDP154A | MH116309 | MH116762 | MH117216 | MH117658 |
| *Poa nemoralis* | YLDP215A | MH116310 | MH116763 | MH117217 | MH117659 |
| *Poa sp* | YLDP137A | MH116311 | MH116764 | MH117218 | MH117660 |
| *Poa trivialis* | YLDP212A | MH116312 | MH116765 | MH117219 | MH117661 |
| *Poa trivialis* | YLDP300A | MH116313 | MH116766 | MH117220 | MH117662 |
| *Polygonatum cirrhifolium* | YLDP157A | — | MH116767 | — | — |
| *Polygonatum cirrhifolium* | YLDP157D | — | MH116768 | — | — |
| *Polygonum glaciale* | YLDP173A | MH116314 | MH116769 | MH117221 | MH117663 |
| *Polygonum glaciale* | YLDP173B | MH116315 | MH116770 | MH117222 | MH117664 |
| *Polygonum nepalense* | YLDP217A | MH116316 | MH116771 | MH117223 | MH117665 |
| *Polygonum nepalense* | YLDP217B | MH116317 | MH116772 | MH117224 | MH117666 |
| *Polygonum nepalense* | YLDP240B | MH116318 | MH116773 | — | MH117667 |
| *Polygonum nepalense* | YLDP293A | MH116319 | MH116774 | MH117225 | MH117668 |
| *Polygonum nepalense* | YLDP293B | MH116320 | MH116775 | MH117226 | MH117669 |
| *Polygonum runcinatum* var. *sinense* | YLDP240A | MH116321 | MH116776 | MH117227 | — |
| *Polygonum runcinatum* | YLDP138A | MH116322 | MH116777 | MH117228 | MH117670 |
| *Polygonum runcinatum* | YLDP138B | MH116323 | MH116778 | MH117229 | MH117671 |
| *Polygonum sinomontanum* | YLDP224A | MH116324 | MH116779 | MH117230 | MH117672 |
| *Polygonum sinomontanum* | YLDP224B | MH116325 | MH116780 | MH117231 | MH117673 |
| *Polygonum suffultum* | YLDP188A | MH116326 | MH116781 | MH117232 | MH117674 |
| *Potentilla lancinata* | YLDP194A | MH116327 | MH116782 | MH117233 | MH117675 |
| *Potentilla lancinata* | YLDP194B | MH116328 | MH116783 | MH117234 | MH117676 |

| | | | | | |
|---|---|---|---|---|---|
| *Potentilla leuconota* | YLDP187A | MH116329 | MH116784 | MH117235 | MH117677 |
| *Potentilla leuconota* | YLDP187B | MH116330 | MH116785 | MH117236 | MH117678 |
| *Primula forrestii* | YLDP094A | MH116331 | MH116786 | MH117237 | MH117679 |
| *Primula forrestii* | YLDP094B | MH116332 | MH116787 | MH117238 | MH117680 |
| *Primula septemloba* | YLDP067A | MH116333 | MH116788 | MH117239 | MH117681 |
| *Primula septemloba* | YLDP067B | MH116334 | MH116789 | MH117240 | MH117682 |
| *Prunella vulgaris* | YLDP054A | MH116335 | — | MH117241 | MH117683 |
| *Prunella vulgaris* | YLDP054B | MH116336 | MH116790 | MH117242 | MH117684 |
| *Prunella vulgaris* | YLDP247A | MH116337 | MH116791 | MH117243 | MH117685 |
| *Prunella vulgaris* | YLDP247B | MH116338 | MH116792 | MH117244 | MH117686 |
| *Pternopetalum delavayi* | YLDP129A | MH116339 | MH116793 | MH117245 | MH117687 |
| *Pternopetalum delavayi* | YLDP129B | MH116340 | MH116794 | MH117246 | MH117688 |
| *Quercus guyavifolia* | YLDP014A | MH116341 | MH116795 | MH117247 | MH117689 |
| *Quercus guyavifolia* | YLDP014C | MH116342 | MH116796 | MH117248 | MH117690 |
| *Quercus guyavifolia* | YLDP014E | MH116343 | MH116797 | MH117249 | MH117691 |
| *Rhodiola yunnanensis* | YLDP167A | MH116344 | MH116798 | MH117250 | MH117692 |
| *Rhodiola yunnanensis* | YLDP167B | MH116345 | MH116799 | MH117251 | MH117693 |
| *Rhododendron decorum* | YLDP062A | MH116346 | MH116800 | MH117252 | MH117694 |
| *Rhododendron decorum* | YLDP062B | MH116347 | — | MH117253 | MH117695 |
| *Rhododendron decorum* | YLDP062C | MH116348 | — | MH117254 | MH117696 |
| *Rhododendron rubiginosum* | YLDP058A | MH116349 | MH116801 | MH117255 | MH117697 |
| *Rhododendron rubiginosum* | YLDP058C | MH116350 | MH116802 | MH117256 | MH117698 |
| *Rhododendron uvariifolium* | YLDP011A | MH116351 | MH116803 | MH117257 | MH117699 |
| *Rhododendron uvariifolium* | YLDP011B | MH116352 | — | MH117258 | MH117700 |
| *Rhododendron uvariifolium* | YLDP011C | MH116353 | — | MH117259 | MH117701 |
| *Rhododendron vernicosum* | YLDP072A | MH116354 | MH116804 | MH117260 | MH117702 |
| *Rhododendron vernicosum* | YLDP072B | MH116355 | MH116805 | MH117261 | MH117703 |
| *Rhododendron yunnanense* | YLDP018A | MH116356 | MH116806 | MH117262 | MH117704 |
| *Rhododendron yunnanense* | YLDP018B | MH116357 | MH116807 | MH117263 | MH117705 |
| *Ribes glaciale* | YLDP035A | MH116358 | MH116808 | MH117264 | MH117706 |
| *Ribes glaciale* | YLDP035B | MH116359 | MH116809 | MH117265 | MH117707 |
| *Ribes glaciale* | YLDP093A | MH116360 | MH116810 | MH117266 | MH117708 |
| *Ribes glaciale* | YLDP093B | MH116361 | MH116811 | — | MH117709 |
| *Ribes himalense* | YLDP273A | MH116362 | MH116812 | MH117267 | MH117710 |
| *Ribes himalense* | YLDP273B | MH116363 | MH116813 | MH117268 | MH117711 |
| *Rodgersia pinnata* | YLDP223A | MH116364 | MH116814 | MH117269 | — |
| *Rodgersia pinnata* | YLDP223B | MH116365 | MH116815 | MH117270 | — |

| | | | | | |
|---|---|---|---|---|---|
| *Rosa sericea* | YLDP061A | MH116366 | MH116816 | MH117271 | MH117712 |
| *Rosa sericea* | YLDP061B | MH116367 | MH116817 | MH117272 | MH117713 |
| *Rosa sertata* | YLDP036A | MH116368 | MH116818 | MH117273 | MH117714 |
| *Rosa sertata* | YLDP156A | MH116369 | MH116819 | MH117274 | MH117715 |
| *Roscoea tibetica* | YLDP155A | MH116370 | MH116820 | MH117275 | MH117716 |
| *Roscoea tibetica* | YLDP155B | MH116371 | — | MH117276 | MH117717 |
| *Rubia membranacea* | YLDP043A | MH116372 | MH116821 | MH117277 | MH117718 |
| *Rubia membranacea* | YLDP043B | MH116373 | MH116822 | MH117278 | MH117719 |
| *Rubia membranacea* | YLDP043C | MH116374 | MH116823 | MH117279 | MH117720 |
| *Rubia membranacea* | YLDP043D | MH116375 | MH116824 | MH117280 | MH117721 |
| *Rubus fockeanus* | YLDP047A | MH116376 | MH116825 | MH117281 | MH117722 |
| *Rubus fockeanus* | YLDP047B | MH116377 | MH116826 | MH117282 | — |
| *Rubus subornatus* | YLDP057A | MH116378 | MH116827 | MH117283 | MH117723 |
| *Rubus subornatus* | YLDP057B | MH116379 | MH116828 | MH117284 | MH117724 |
| *Rubus subornatus* | YLDP057C | MH116380 | MH116829 | MH117285 | MH117725 |
| *Rumex nepalensis* | YLDP184A | MH116381 | MH116830 | MH117286 | MH117726 |
| *Rumex nepalensis* | YLDP184C | MH116382 | MH116831 | MH117287 | MH117727 |
| *Sabia yunnanensis* | YLDP030A | MH116383 | MH116832 | MH117288 | MH117728 |
| *Sabia yunnanensis* | YLDP030B | MH116384 | MH116833 | MH117289 | MH117729 |
| *Sabia yunnanensis* | YLDP097A | MH116385 | MH116834 | MH117290 | MH117730 |
| *Sabia yunnanensis* | YLDP097B | MH116386 | MH116835 | MH117291 | MH117731 |
| *Salix balfouriana* | YLDP268A | MH116387 | MH116836 | MH117292 | MH117732 |
| *Salix delavayana* | YLDP277A | MH116388 | MH116837 | MH117293 | MH117733 |
| *Salix delavayana* | YLDP277B | MH116389 | MH116838 | MH117294 | MH117734 |
| *Salix delavayana* | YLDP277D | MH116390 | MH116839 | MH117295 | MH117735 |
| *Salix dibapha* | YLDP277E | MH116391 | MH116840 | MH117296 | MH117736 |
| *Salix eriostachya* | YLDP277C | MH116392 | MH116841 | MH117297 | MH117737 |
| *Salvia bifidocalyx* | YLDP220A | MH116393 | MH116842 | MH117298 | MH117738 |
| *Salvia bifidocalyx* | YLDP220B | MH116394 | MH116843 | MH117299 | MH117739 |
| *Sambucus javanica* | YLDP192A | MH116395 | MH116844 | MH117300 | MH117740 |
| *Sambucus javanica* | YLDP192B | MH116396 | MH116845 | MH117301 | MH117741 |
| *Sambucus javanica* | YLDP192C | MH116397 | MH116846 | MH117302 | MH117742 |
| *Sanicula hacquetioides* | YLDP121A | MH116398 | MH116847 | MH117303 | MH117743 |
| *Sanicula hacquetioides* | YLDP121B | MH116399 | MH116848 | MH117304 | MH117744 |
| *Saussurea peduncularis* | YLDP235A | MH116400 | MH116849 | MH117305 | MH117745 |
| *Saussurea peduncularis* | YLDP235B | MH116401 | MH116850 | MH117306 | MH117746 |
| *Saxifraga diversifolia* | YLDP048A | MH116402 | MH116851 | MH117307 | MH117747 |

| | | | | | |
|---|---|---|---|---|---|
| *Saxifraga diversifolia* | YLDP048B | MH116403 | MH116852 | MH117308 | MH117748 |
| *Saxifraga diversifolia* | YLDP048C | MH116404 | MH116853 | MH117309 | MH117749 |
| *Saxifraga oreophila* | YLDP242A | MH116405 | MH116854 | MH117310 | MH117750 |
| *Saxifraga oreophila* | YLDP242B | MH116406 | MH116855 | MH117311 | MH117751 |
| *Saxifraga rufescens* | YLDP197B | MH116407 | MH116856 | MH117312 | MH117752 |
| *Saxifraga rufescens* | YLDP197D | MH116408 | MH116857 | MH117313 | MH117753 |
| *Saxifraga strigosa* | YLDP232A | MH116409 | MH116858 | MH117314 | MH117754 |
| *Saxifraga strigosa* | YLDP232D | MH116410 | MH116859 | MH117315 | — |
| *Schisandra sphaerandra* | YLDP226A | MH116411 | MH116860 | MH117316 | MH117755 |
| *Schisandra sphaerandra* | YLDP226B | MH116412 | MH116861 | MH117317 | MH117756 |
| *Sedum multicaule* | YLDP040A | MH116413 | — | MH117318 | MH117757 |
| *Sedum multicaule* | YLDP040B | MH116414 | MH116862 | MH117319 | MH117758 |
| *Sedum multicaule* | YLDP040C | MH116415 | MH116863 | MH117320 | MH117759 |
| *Smilax menispermoidea* | YLDP092A | MH116416 | MH116864 | MH117321 | — |
| *Smilax menispermoidea* | YLDP092B | MH116417 | MH116865 | MH117322 | — |
| *Sorbus coronata* | YLDP078A | MH116418 | MH116866 | — | MH117760 |
| *Sorbus coronata* | YLDP078C | MH116419 | MH116867 | — | MH117761 |
| *Sorbus hupehensis* | YLDP074A | MH116420 | MH116868 | MH117323 | MH117762 |
| *Sorbus hupehensis* | YLDP074B | MH116421 | MH116869 | — | MH117763 |
| *Sorbus prattii* | YLDP005A | MH116422 | MH116870 | MH117324 | MH117764 |
| *Sorbus prattii* | YLDP006A | MH116423 | MH116871 | — | MH117765 |
| *Sorbus prattii* | YLDP006B | MH116424 | MH116872 | MH117325 | MH117766 |
| *Sorbus prattii* | YLDP279A | MH116425 | MH116873 | MH117326 | MH117767 |
| *Sorbus rufopilosa* | YLDP140A | MH116426 | MH116874 | MH117327 | — |
| *Sorbus rufopilosa* | YLDP140B | MH116427 | MH116875 | — | MH117768 |
| *Spiraea schneideriana* | YLDP063A | MH116428 | MH116876 | MH117328 | MH117769 |
| *Spiraea schneideriana* | YLDP063B | MH116429 | MH116877 | MH117329 | MH117770 |
| *Stachyurus chinensis* var. *brachystachyus* | YLDP141A | MH116430 | MH116878 | MH117330 | MH117771 |
| *Stachyurus chinensis* var. *brachystachyus* | YLDP141B | MH116431 | MH116879 | MH117331 | MH117772 |
| *Stellaria vestita* | YLDP071A | — | MH116880 | — | — |
| *Stellaria vestita* | YLDP071B | MH116432 | MH116881 | MH117332 | MH117773 |
| *Stellaria vestita* | YLDP071D | MH116433 | MH116882 | MH117333 | MH117774 |
| *Stellaria vestita* | YLDP071E | MH116434 | MH116883 | MH117334 | MH117775 |
| *Stellaria vestita* | YLDP127A | MH116435 | MH116884 | MH117335 | MH117776 |
| *Stellaria vestita* | YLDP127B | — | MH116885 | — | — |
| *Swertia macrosperma* | YLDP023A | MH116436 | MH116886 | MH117336 | MH117777 |
| *Swertia macrosperma* | YLDP023B | MH116437 | MH116887 | MH117337 | MH117778 |

| | | | | | |
|---|---|---|---|---|---|
| *Swertia macrosperma* | YLDP249A | MH116438 | MH116888 | MH117338 | MH117779 |
| *Swertia macrosperma* | YLDP249B | MH116439 | MH116889 | MH117339 | MH117780 |
| *Synotis erythropappa* | YLDP026A | MH116440 | MH116890 | MH117340 | MH117781 |
| *Synotis erythropappa* | YLDP026B | MH116441 | MH116891 | MH117341 | — |
| *Synotis erythropappa* | YLDP026D | MH116442 | MH116892 | MH117342 | MH117782 |
| *Syringa yunnanensis* | YLDP079A | MH116443 | MH116893 | MH117343 | MH117783 |
| *Syringa yunnanensis* | YLDP079B | MH116444 | MH116894 | MH117344 | MH117784 |
| *Taraxacum dasypodum* | YLDP119A | MH116445 | MH116895 | MH117345 | MH117785 |
| *Taraxacum dasypodum* | YLDP119B | MH116446 | MH116896 | MH117346 | MH117786 |
| *Taraxacum dasypodum* | YLDP119C | MH116447 | MH116897 | MH117347 | MH117787 |
| *Taxillus delavayi* | YLDP032A | MH116448 | MH116898 | MH117348 | MH117788 |
| *Taxillus delavayi* | YLDP032B | MH116449 | MH116899 | MH117349 | MH117789 |
| *Taxillus delavayi* | YLDP032C | MH116450 | MH116900 | MH117350 | MH117790 |
| *Thalictrum delavayi* | YLDP052A | MH116451 | MH116901 | MH117351 | MH117791 |
| *Thalictrum delavayi* | YLDP052B | MH116452 | MH116902 | MH117352 | MH117792 |
| *Thalictrum delavayi* | YLDP052C | MH116453 | MH116903 | — | MH117793 |
| *Tilia chinensis* | YLDP080A | MH116454 | MH116904 | MH117353 | — |
| *Tripterospermum volubile* | YLDP204A | MH116455 | MH116905 | MH117354 | MH117794 |
| *Tripterospermum volubile* | YLDP204B | MH116456 | MH116906 | MH117355 | MH117795 |
| *Valeriana hardwickii* | YLDP257A | MH116457 | MH116907 | MH117356 | MH117796 |
| *Valeriana hardwickii* | YLDP257B | MH116458 | MH116908 | MH117357 | MH117797 |
| *Veronica piroliformis* | YLDP132A | MH116459 | MH116909 | MH117358 | MH117798 |
| *Veronica piroliformis* | YLDP132C | MH116460 | MH116910 | MH117359 | MH117799 |
| *Viburnum betulifolium* | YLDP019A | MH116461 | MH116911 | MH117360 | — |
| *Viburnum betulifolium* | YLDP019B | MH116462 | MH116912 | MH117361 | — |
| *Viburnum betulifolium* | YLDP019C | MH116463 | MH116913 | MH117362 | MH117800 |
| *Viola biflora* | YLDP158A | MH116464 | MH116914 | MH117363 | MH117801 |
| *Viola biflora* | YLDP158B | MH116465 | MH116915 | MH117364 | MH117802 |
| *Viola moupinensis* | YLDP120A | MH116466 | MH116916 | MH117365 | MH117803 |
| *Viola moupinensis* | YLDP120B | MH116467 | MH116917 | MH117366 | MH117804 |
| *Viola urophylla* | YLDP099A | MH116468 | MH116918 | MH117367 | MH117805 |
| *Viola urophylla* | YLDP099B | MH116469 | MH116919 | MH117368 | MH117806 |
| *Youngia paleacea* | YLDP234B | MH116470 | MH116920 | — | MH117807 |
| *Youngia paleacea* | YLDP234C | MH116471 | MH116921 | MH117369 | MH117808 |
| *Youngia paleacea* | YLDP239A | MH116472 | MH116922 | MH117370 | — |
| *Youngia paleacea* | YLDP239B | MH116473 | MH116923 | MH117371 | MH117809 |
| *Abies forrestii* | YLDP003A | MH116474 | MH116924 | MH117372 | MH117810 |

| | | | | | |
|---|---|---|---|---|---|
| *Abies forrestii* | YLDP003B | MH116475 | MH116925 | MH117373 | MH117811 |
| *Abies forrestii* | YLDP101A | MH116476 | MH116926 | MH117374 | MH117812 |
| *Abies forrestii* | YLDP101B | MH116477 | MH116927 | MH117375 | MH117813 |
| *Abies forrestii* | YLDP103A | MH116478 | MH116928 | MH117376 | MH117814 |
| *Juniperus squamata* | YLDP109A | MH116479 | MH116929 | MH117377 | MH117815 |
| *Juniperus squamata* | YLDP109B | MH116480 | MH116930 | MH117378 | MH117816 |
| *Picea likiangensis* | YLDP021A | MH116481 | MH116931 | MH117379 | MH117817 |
| *Picea likiangensis* | YLDP021B | MH116482 | MH116932 | MH117380 | MH117818 |
| *Pinus armandii* | YLDP059A | MH116483 | MH116933 | MH117381 | MH117819 |
| *Pinus armandii* | YLDP059B | MH116484 | MH116934 | MH117382 | MH117820 |
| *Taxus florinii* | YLDP046A | MH116485 | MH116935 | MH117383 | MH117821 |
| *Taxus florinii* | YLDP046B | MH116486 | MH116936 | MH117384 | MH117822 |
| *Tsuga dumosa* | YLDP102B | MH116487 | MH116937 | MH117385 | — |
| *Tsuga dumosa* | YLDP102C | MH116488 | MH116938 | MH117386 | MH117823 |

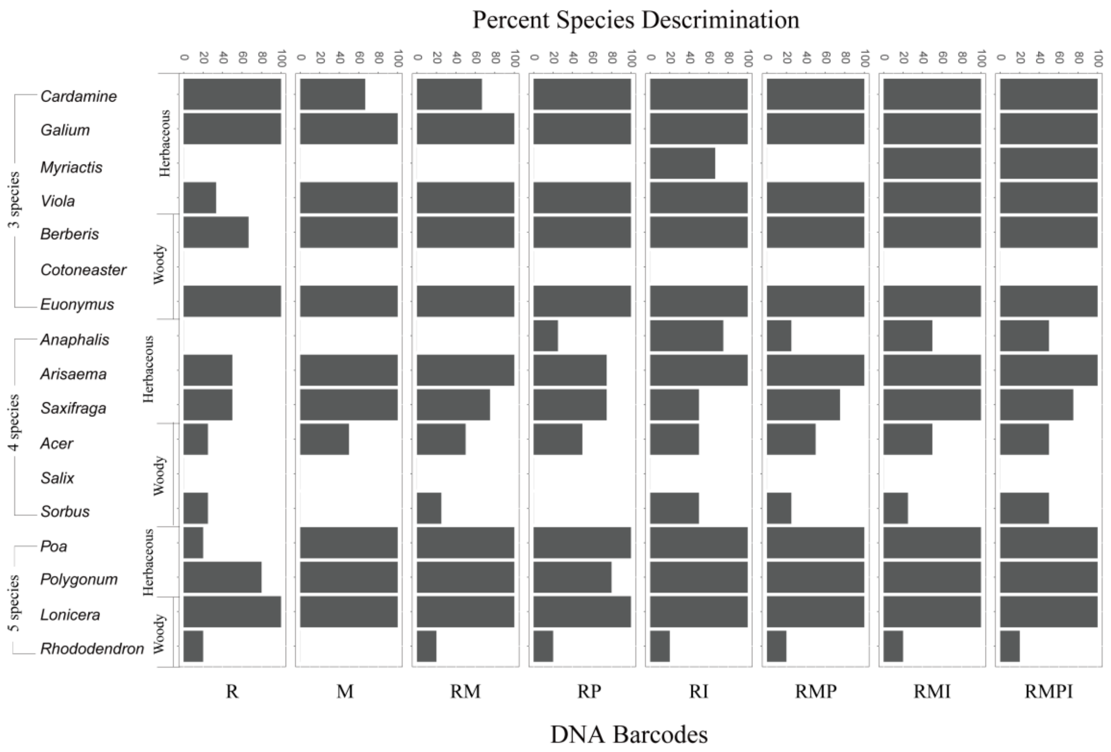


Figure S1. Species discrimination success in genera with greater than three species collected from a high elevation subalpine forest dynamics plot on Yulong Mountain, Northwestern Yunnan, China. We assessed variation in percentage species discrimination for *rbcL* [R] and *matK* [M] barcodes separately, and in combination (*rbcL* + *matK* [RM], *rbcL* + *trnH-psbA* [RP], *rbcL* + ITS [RI], *rbcL* + *matK* + *trnH-psbA* [RMP], *rbcL* + *matK* + ITS [RMI], *rbcL* + *matK* + *trnH-psbA* + ITS [RMPI]. None of the species in *Salix* and *Cotoneaster* were resolved by any barcode or barcode combination. *Myriactis*, *Anaphalis*, *Rhododendron*, *Sorbus*, and *Acer* also showed low rates for percentage species discrimination.

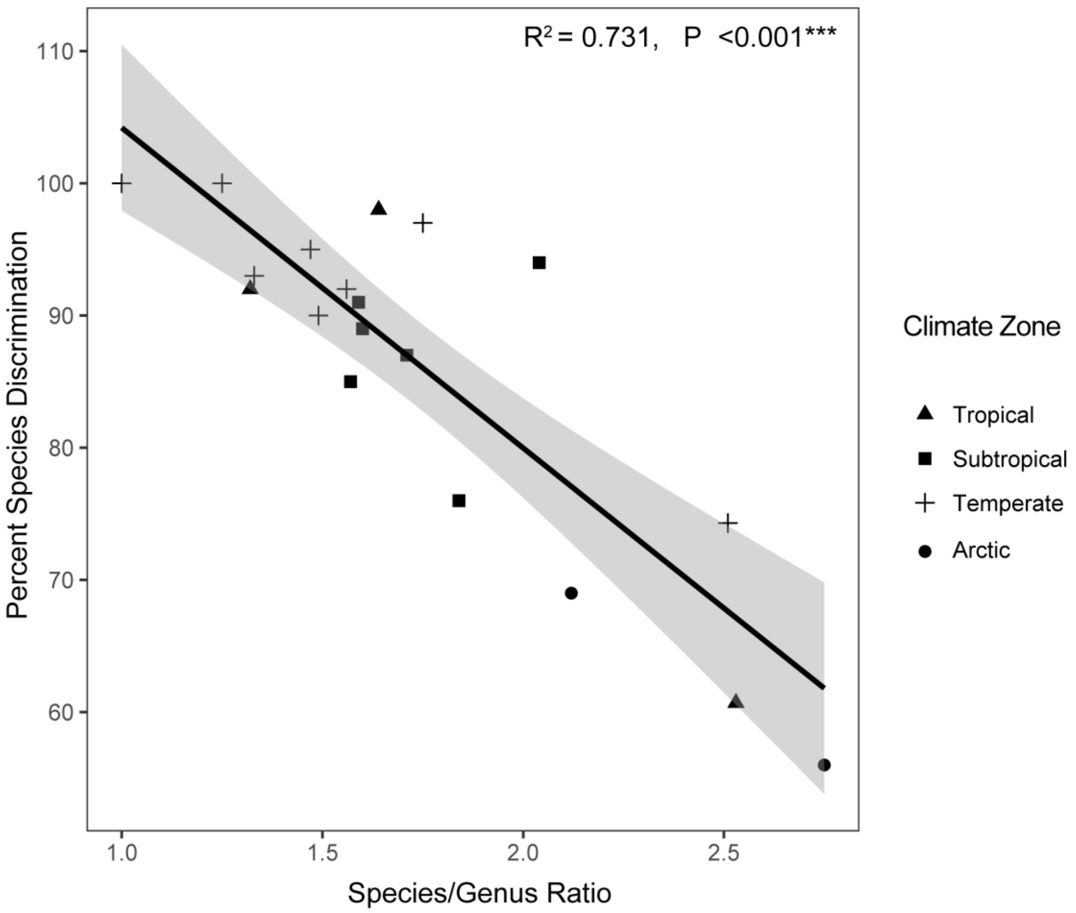


Figure S2. Relationships between percent species discrimination and species/genus ratio summarized from plot and floristic barcoding studies as listed in Table 3. The level of species discrimination is significantly negatively correlated with species/genus ratio.
